# Zinc effects on bacteria: insights from *Escherichia coli* by multi-omics approach

**DOI:** 10.1128/msystems.00733-23

**Published:** 2023-10-31

**Authors:** Martin Rihacek, Ludmila Kosaristanova, Tatiana Fialova, Michaela Kuthanova, Ales Eichmeier, Eliska Hakalova, Martin Cerny, Miroslav Berka, Jana Palkovicova, Monika Dolejska, Pavel Svec, Vojtech Adam, Ludek Zurek, Kristyna Cihalova

**Affiliations:** 1 Department of Chemistry and Biochemistry, Faculty of AgriSciences, Mendel University in Brno, Brno, Czechia; 2 Faculty of Horticulture, Mendeleum—Institute of Genetics, Mendel University in Brno, Brno, Czechia; 3 Department of Molecular Biology and Radiobiology, Faculty of AgriSciences Mendel University in Brno, Brno, Czechia; 4 Faculty of Medicine in Pilsen, Biomedical Center, Charles University, Pilsen, Czechia; 5 Central European Institute of Technology, University of Veterinary Sciences Brno, Brno, Czechia; 6 Department of Biology and Wildlife Diseases, Faculty of Veterinary Hygiene and Ecology, University of Veterinary Sciences Brno, Brno, Czechia; 7 Department of Clinical Microbiology and Immunology, Institute of Laboratory Medicine, The University Hospital Brno, Brno, Czechia; Marquette University, Milwaukee, Wisconsi, USA

**Keywords:** zinc, zinc oxide, nanoparticles, phenotype, antimicrobial resistance, virulence, genome, transcriptome, proteome

## Abstract

In this study, we investigated the impact of zinc oxide (ZnO) and zinc oxide nanoparticles (ZnONPs) on *Escherichia coli*. These compounds are commonly used as fertilizers and feed additives and may have unintended consequences on bacteria. We conducted phenotypic and multi-omics analyses of *E. coli* exposed to sub-lethal concentrations of ZnO and ZnONPs for 40 sub-culturings and 20 sub-culturings with zinc followed by 20 sub-culturings without zinc in an attempt to reverse zinc effects. An extended treatment with ZnO for 40 sub-culturings had the greatest impact leading to bacterial resistance to aminoglycosides, cephalosporins, and sulfonamides while the treatment with ZnONPs for 40 sub-culturings led to the elevated MIC to chloramphenicol only. Cells exposed to these treatments were thicker and had retarded growth in elevated temperatures. Importantly, zinc withdrawal reversed most phenotypic changes. Zinc exposure caused dramatic changes in cell transcripts and proteins with a role in antibiotic response, heat stress, growth regulation, cell shape, and biofilm formation. An extended zinc exposure led to overall major alterations in *E. coli* biology and resulted in multi-drug resistance.

**IMPORTANCE:**

A long-term exposure of bacteria to zinc oxide and zinc oxide nanoparticles leads to major alterations in bacterial morphology and physiology. These included biochemical and physiological processes promoting the emergence of strains with multi-drug resistance and virulence traits. After the removal of zinc pressure, bacterial phenotype reversed back to the original state; however, certain changes at the genomic, transcriptomic, and proteomic level remained. Why is this important? The extensive and intensive use of supplements in animal feed effects the intestinal microbiota of livestock and this may negatively impact the health of animals and people. Therefore, it is crucial to understand and monitor the impact of feed supplements on intestinal microorganisms in order to adequately assess and prevent potential health risks.

## INTRODUCTION

Environmental pollution is one of the most serious problems confronting modern society (1). Heavy metals are commonly present in the environment; however, human activities resulted in heavy metals accumulation with negative impacts on the environment and living organisms (2). It has been demonstrated that the soil may contain high concentrations of zinc (Zn) due to its heavy use in agriculture as feed supplements, fertilizers, and pesticides (3, 4). Since the European Union ban on the use of antimicrobials for animal growth promotion in 2006, zinc has been used in the form of zinc oxide (ZnO) in livestock to increase animal growth and for prevention of bacterial infections (5, 6). For example, ZnO has been commonly used around the world in diets of weaning piglets in high doses (2,000 mg–3,000 mg/kg feed) to reduce the incidence of diarrhea and to improve piglet growth (7, 8). Due to the resulting heavy contamination of the environment, as of June 2022, the current maximum level of zinc allowed for livestock is 150 mg/kg of the complete feed (9). Consequently, heavy metals are used in the form of new synthetic compounds, including nanoparticles.

Zinc oxide nanoparticles (ZnONPs) belong to the third most produced nanomaterial worldwide (>60,000 tons/year) with significant ecological footprints (10). Despite the reduced concentration of zinc in livestock feeding, many studies have reported that the presence of a very low metal, antibiotic, or biocide concentration plays an essential role in the selection, proliferation, and maintenance of existing resistant mutants (11–14). Furthermore, a long-term exposure of bacteria to sub-lethal doses of environmental pollutants may lead to the evolution, horizontal transfer, and spread of antimicrobial resistance genes (11) as well as increased prevalence and spread of virulence traits (15). Cellular changes at the genomic, transcriptomic, and proteomic level can be manifested in the bacterial phenotype, which can then significantly disturb the balance of the microbiome, including that of the mammalian gastro-intestinal tract (15). It is known that mutations in genes involved in transcription and translation, structural genes, and those in membrane transport are behind the mechanism of metal-induced resistance to antibiotics (16). Typically, selection pressure results in the ability of the resistant bacterial subpopulation to outgrow the susceptible ones, and after pressure withdrawal, resistant cells lose acquired resistance traits in order to eliminate fitness costs (17). However, this is not always the case (18). The effect of sub-lethal doses of antibiotics and heavy metals on gene and protein regulation is variable and can manifest as co-/cross-resistance to antibiotics and metals, but also as many other alterations (19, 20). Changes in bacteria are very fast compared to that of higher organisms and therefore dangerous with respect to multi-drug resistance and virulence (20). Even if these manifestations are minor at first, for example, changes in bacterial growth, cell shape, quorum sensing, biofilm formation, and the shift of the minimum inhibitory concentration, their effect can eventually lead to a phenotype causing life-threatening infections (21–25). It has been shown that plasmids and other mobile genetic elements can carry genes for resistance to antibiotics and heavy metals, and the induction of a gene coding antibiotic resistance can also lead to the induction of a gene for resistance to zinc and vice versa (26). A heavy metal exposure, apart from triggering co-selection through co-resistance and cross-resistance, can enhance tolerance to antibiotics by affecting expression of antimicrobial resistance genes (19, 27). On the other hand, ZnO and ZnONPs can damage bacteria by releasing Zn2+ ions causing cell membrane disruption (28) and by induction of reactive oxygen species (ROS) (29).

This study focused on the phenotypic and multi-omics analyses of *Escherichia coli* exposed to sub-lethal concentrations of ZnO and ZnONPs for either 40 sub-culturing (ZnO40 and ZnONPs40) or for 20 sub-culturing with Zn followed by 20 sub-culturing without Zn (ZnO20+20 and ZnONPs20+20) in an attempt to reverse zinc effects. The main focus of the study was to analyze the changes in bacterial cells from perspectives of antimicrobial resistance and virulence.

## RESULTS

### Antibiotic susceptibility

The study monitored the effects of sub-inhibitory concentrations of ZnO and ZnONPs (the first concentration at which bacteria grew below the minimum inhibitory concentration) on *E. coli* over 40 sub-cultures. It was observed that the sub-inhibitory concentrations increased progressively up to 4.0 g/L at the 23rd sub-culture for ZnO and the 20th sub-culture for ZnONPs. Afterward, the concentrations fluctuated, eventually reaching the final concentration of 1.0 g/L at the 40th sub-culture (Table 1).

**TABLE 1:** Sub-inhibitory concentrations of ZnO/ZnONPs used in individual sub-cultures of *E. coli*

Sub-inhibitory concentrations of ZnO/ZnONPs (g/L)																				
Sub-cultures	1	2	3	4	5	6	7	8	9	10	11	12	13	14	15	16	17	18	19	20
ZnO	0.5	1	0.5	0.5	1	0.5	1	1	2	2	2	2	2	2	2	2	2	2	2	2
ZnONPs	0.5	1	0.5	0.5	1	1	1	1	2	2	2	1	2	1	1	2	2	2	2	4
Sub-cultures	21	22	23	24	25	26	27	28	29	30	31	32	33	34	35	36	37	38	39	40
ZnO	2	2	4	2	1	2	4	2	4	2	2	2	1	1	2	2	1	1	2	1
ZnONPs	4	4	2	4	2	2	2	2	2	2	2	2	2	1	1	1	1	1	1	1

**Fig 1:**
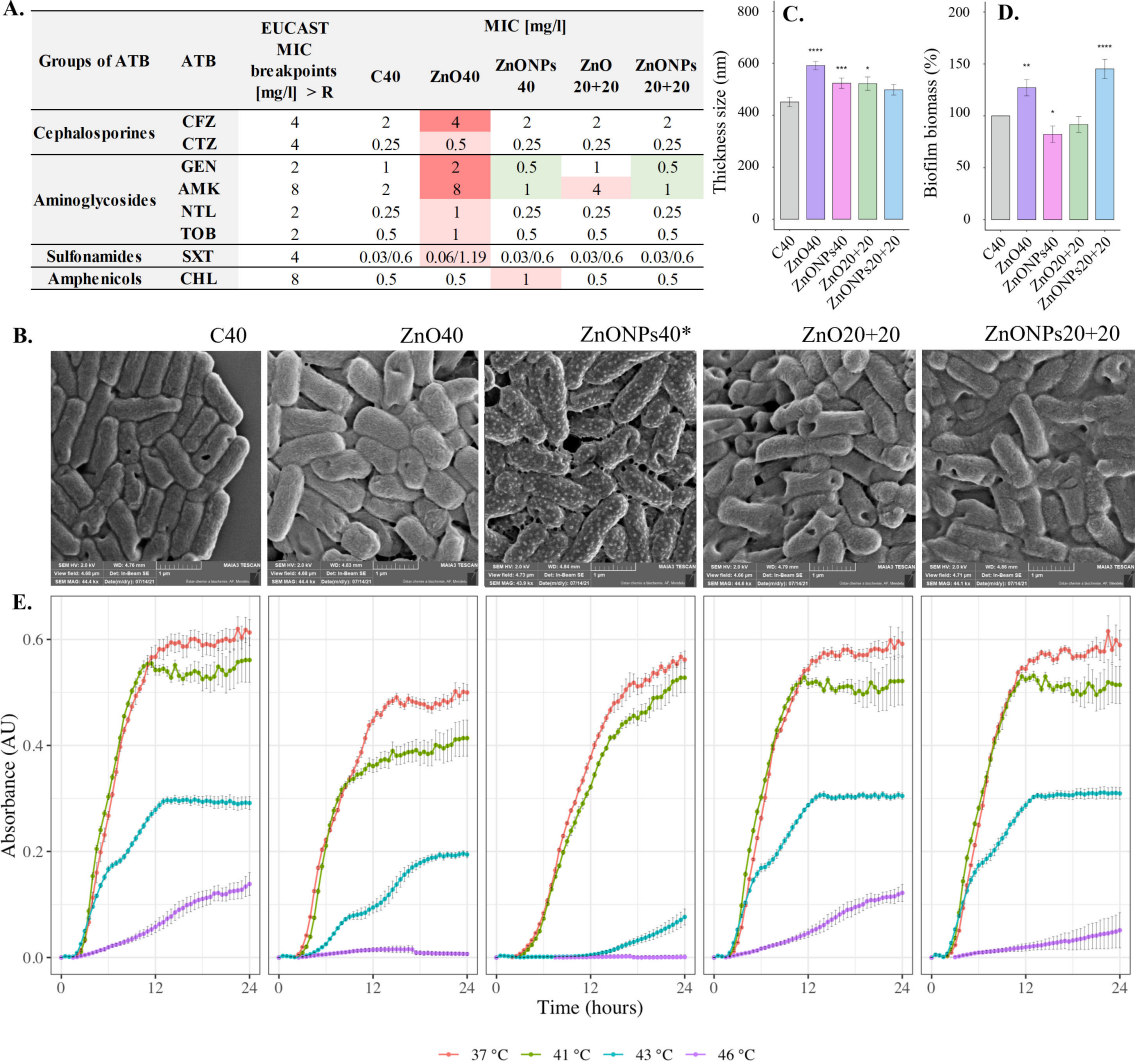
Phenotypicdefine profile of *E. coli* after various treatments: (**A)** Changes in minimum inhibitory concentration of antibiotics: CFZ, CTZ, GEN, AMK, NTL, TOB, SXT, and CHL. The dark red color indicates the breakpoint value has been reached, the light red color indicates the increasing of MIC value after treatment in comparison to C40 without reaching the breakpoint. The light green color depicts decreased values of MIC after treatment in comparison to that of C40; (**B)** cell morphology after various treatments; (**C)** cell thickness after various treatments; (**D)** biofilm biomass formation after various treatments; (**E)** bacterial growth at different temperatures (37°C, 41°C, 43°C, 46°C) after various treatments. The asterisks in C and D represent the significance accoding to obtained p-value: no * presence represents no sinificance, *p < 0.05, **p < 0.01, ***p < 0.001, and ****p < 0.0001. *In figure B ZnONPs40 represents a crystallization on the cells is an artifact during cell dehydration.

A total of 24 antibiotics from different groups including penicillins, monobactams, cephalosporins, aminoglycosides, sulfonamides, fluoroquinolones, carbapenems, tetracyclines, amphenicols, and antimicrobial peptides were tested (Table S1B). The MIC was determined after 40th sub-cultures for all treatments (ZnO40, ZnONPs40, ZnO20+20, and ZnONPs20+20) and compared to that of the untreated cells (C40). The most frequent and significant changes in MIC critical breakpoints, signifying bacterial resistance above these concentrations, according to the European Committee on Antimicrobial Susceptibility Testing (EUCAST) (30) were detected after 40 sub-cultures of *E. coli* with ZnO (ZnO40) to cefazolin (CFZ), gentamicin (GEN), and amikacin (AMK) (Fig. 1A). The same MIC of these antibiotics was found at the 20th sub-culture already. This treatment also increased concentrations at the limit of the critical breakpoint for CFZ, GEN, and AMK (4, 2, and 8 mg/L, respectively). Increases in MIC were also detected in the ZnO40 treatment for ceftazidime (CTZ), netilmicin (NTL), tobramycin (TOB), and trimethoprim/sulfamethoxazole (SXT), although the critical breakpoints were not reached (Fig. 1A). In the ZnO20+20 treatment, a decrease in the MIC was observed compared to that of 20th and 40th sub-cultures exposed to ZnO. Only MIC for AMK (4 mg/L) remained elevated in comparison to that of the C40. Extended exposure of *E. coli* to ZnONPs increased the MIC only for chloramphenicol (CHL), and this did not reach the critical breakpoint (8.0 mg/L). In fact, exposure of *E. coli* to ZnONPs decreased the MIC for aminoglycosides GEN and AMK (0.5 mg/L and 1.0 mg/L) in both sub-cultures (20th and 40th) compared to that of C40. This decrease was maintained even after the elimination of ZnONPs for 20 sub-cultures (ZnONPs20+20) (Fig. 1A; Table S1B). This analysis confirmed our hypothesis that the sub-lethal doses of zinc in forms of ZnO and ZnONPs induce resistance to some antibiotics.

### Bacterial morphology

Another zinc-dependent phenotype alteration was detected in the bacterial cell size. Extended exposure of *E. coli* to ZnO and ZnONPs resulted in the significantly thicker cells (*P*-value < 0.05) when compared to that of C40 (Fig. 1B and C). The average cell width was 591 ± 16 nm (ZnO40) and 568 ± 15 nm (ZnONPs40) while the cell width of the control was 451 ± 18 nm (Fig. 1C). This trend was not seen in the other treatments. Removal of zinc after 20th sub-culturing resulted only in a slight increase in the cell width: 522 ± 26 nm (ZnO20 + 20) and 498 ± 20 nm (ZnONPs20 + 20) (Fig. 1C; Fig. S1A through E).

### Biofilm formation

Zinc plays a role in virulence; thus, our study focused on the ability of zinc-treated *E. coli* to form biofilm. The Alamar Blue assay results revealed a difference in bacterial biofilm formation in response to zinc treatments (Fig. 1D). The biofilm formation is shown in percentages with 100 ± 6% for C40. Biofilm formation increased (*P*-value <0.05) under ZnO40 and ZnONPs20+20 with values of 131 ± 5% and 150 ± 7%, respectively. In contrast, a decrease in biofilm formation (*P*-value <0.05) was observed in the treatment ZnONPs40 (85 ± 5%) (Fig. 1D).

### Bacterial growth

Measurements of bacterial growth at temperatures 37°C, 41°C, 43°C, and 46°C revealed differences among *E. coli* strains (Fig. 1E). As the temperature increased, the growth of *E. coli* decreased across all tested strains. At temperature 43°C, bacteria exposed to ZnO40 and ZnONPs40 showed weak or no growth, compared to that of C40, ZnO20+20, and ZnONPs20+20. In general, the growth of ZnO20+20 and ZnONPs20+20 was similar to that of C40 while the extended treatments (ZnO40 and ZnONPs40) negatively impacted bacterial growth under all tested temperatures (Fig. 1E).

### Genomic analysis

**Fig 2:**
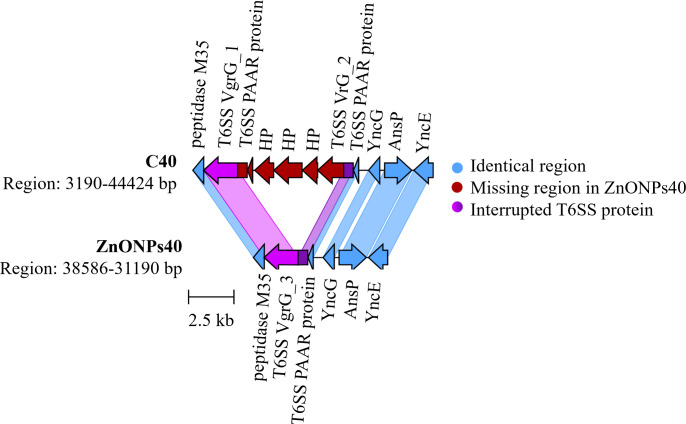
Representation of the T6SS PAAR region in genome and deletion of three genes coding hypothetical proteins in ZnONPs40 compared to that of untreated control C40.

Genetic characterization of *E. coli* ATCC 25922 under different treatments is shown in Table S2A. Single nucleotide polymorphism mutations in genes occurred in all treatments (Table 2). Mutations occurred in genes which products can respond to ZnO and ZnONPs and affect stress response (*rpoS* and *cpxA* for ZnO40, *rpoS* for ZnONPs40, *hns* for ZnONPs20+20), oxidoreductase activity (*qorB* for ZnO20+20), and metal binding activity (*topA* for ZnO40). The number of single-nucleotide polymorphism (SNP) mutations in genes varied across treatments, with ZnO40 having the highest number of SNPs, followed by ZnONPs40, ZnO20+20, and ZnONPs20+20 (Table 2). Only one SNP mutation in the *rpoS* gene was found in two different treatments: ZnO40 and ZnONPs40. An extended exposure to ZnO and ZnONPs (ZnO40 and ZnONPs40) resulted in nucleotide substitution in *rpoS*. In the parent *E. coli* ATCC 25922 and the ceased treatments (C40, ZnO20+20, ZnONPs20+20), the codon in the position of the mutation (49777) is the stop codon. After an extended exposure to zinc (ZnO40 and ZnONPs40), the mutation was not detected. Other genes with SNPs coded for membrane-bound lytic murein transglycosylase MltF, IS1 transposase, and hypothetical protein (ZnO40), tRNA-Leu, two SNP mutations in phosphatidate cytidylyltransferase CdsA (ZnONPs40), ATP-dependent RNA helicase DeaD, and NADPH-dependent assimilatory sulfite reductase flavoprotein subunit (ZnO20+20). In ZnONPs40, 5,584 bp long deletion was detected (Fig. 2). Two type VI secretion system (T6SS) proteins VgrG (VgrG_1 and VgrG_2) of a slightly different length (2,406 bp and 1,959 bp) with a similar sequence at the 5´ end of the proteins flanked this region. Seemingly, this deletion was generated due to a recombination in the similar regions of these proteins, probably because of the DNA damage and the subsequent repair. This resulted in a formation of the hybrid T6SS protein VgrG_3 and deletion of the T6SS PAAR protein and three hypothetical proteins (Fig. 2). All intergenic mutations are listed in the Table S2B.

**TABLE 2:** Single nucleotide polymorphism mutations in genes developed in all treated strains with selected GO terms for biological processes

Treated strain	Mutation type^*a*^	Original codon/base (AA)^*b*^	Alternative codon/base (AA)^*c*^	SNP location on contig^*d*^	Genes	Affected product^*e*^	Product location on contig^*f*^	GO terms BP^*g*^
ZnO40	SNP	ACC (T)	CCC (P)	30009	*mltF*	Membrane-bound lytic murein transglycosylase MltF	30162–28606	Cell wall macromolecule catabolic process, cell wall organization, peptidoglycan catabolic process
	SNP	TAG (*)	CAG (Q)	49777	*rpoS*	RNA polymerase sigma factor RpoS	49951–49775	Positive regulation of single-species biofilm formation on inanimate substrate, regulation of cell motility, regulation of cellular response to heat and oxidative stress, regulation of response to salt stress
	SNP	CTG (L)	CTT (L)	88393	*topA*	Type I DNA topoisomerase	89784–87187	Metal ion binding
	SNP	GTT (V)	ATT (I)	50610	*ins1*	IS1 transposase	50502–51195	DNA recombination, transposition
	SNP	CTG (L)	ATT (I)	10048	*cpxA*	Envelope stress sensor histidine kinase CpxA	10565–9192	Cell adhesion involved in biofilm formation, cellular response to cell envelope stress
	SNP	AAC (N)	AAT (N)	429		Hypothetical protein	620–1	
ZnONPs40	SNP	GTA (V)	GTC (V)	99400	*tRNA-Leu*	tRNA-Leu	99317–99403	Protein biosynthesis
	SNP	TAG (*)	CAG (Q)	49777	*rpoS*	RNA polymerase sigma factor RpoS	49951–49775	Positive regulation of single-species biofilm formation on inanimate substrate, regulation of cell motility, regulation of cellular response to heat and oxidative stress, regulation of response to salt stress
	SNP	TTA (L)	TCA (S)	19732	*cdsA*	Phosphatidate cytidylyltransferase CdsA	20417–19560	Lipid biosynthesis and metabolism, phospholipid biosynthesis and metabolism
	SNP	TTC (F)	GTC (V)	20336	*cdsA*	Phosphatidate cytidylyltransferase CdsA	20417–19560	Lipid biosynthesis and metabolism, phospholipid biosynthesis and metabolism
	Deletion		−5584 bp		*vgrG_1–vgrG_2*	T6SS protein VgrG (two copies), T6SS PAAR protein, hypothetical proteins		Cytolysis, bacteriocin, hydrolase
ZnO20 + 20	SNP	GGC (G)	GAC (D)	115147	*deaD*	ATP-dependent RNA helicase DeaD	113613–115502	Processes at low temperatures, cold-shock degradosome with Rnase E
	SNP	GGT (G)	TGT (C)	63243	*cysJ*	NADPH-dependent assimilatory sulfite reductase flavoprotein subunit CysJ	64878–63079	FMN binding, sulfite reductase (NADPH) activity, cysteine biosynthesis
	SNP	GTG (V)	ATG (M)	49967	*qorB*	NAD(*P*)H:quinone oxidoreductase QorB	50507–49647	Oxidoreductase activity
ZnONPs20 + 20	SNP	ATT (I)	GTT (V)	126557	*hns*	DNA-binding transcriptional regulator H-NS		Stress response

^*a*^ Detected mutation type where SNP represents single nucleotide polymorphism.

^*b*^ Original codon (if present in coding sequence) or base (if intergenic) in C40 with amino acid (AA) change for coding sequences.

^*c*^ Alternative codon (if present in coding sequence) or base (if intergenic) in a treated strain with AA change for coding sequences.

^*d*^ Location of SNP or product in short-read assembly.

^*e*^ Product(s) affected by the mutation.

^*f*^ Product(s) location on contig.

^*g*^ Biological process in GO terms specified in UniProt database, accessed 2 March 2023.

### Transcriptomic analysis

**Fig 3:**
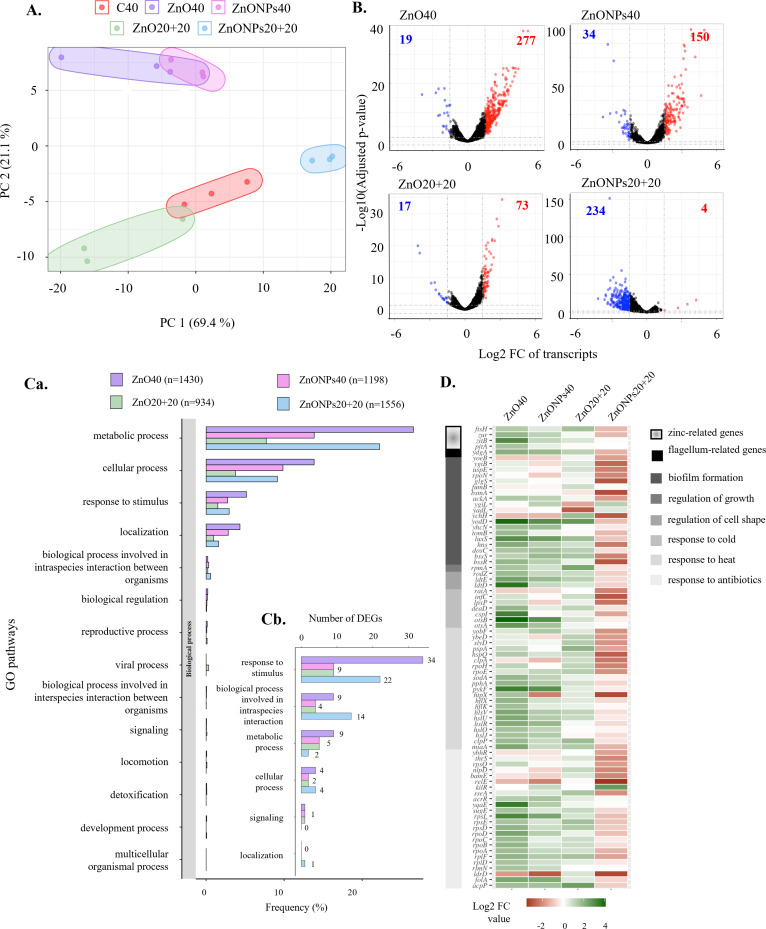
Transcriptomic analysis: (**A)** PCA plots of biological triplicates for various treatments. (**B)** Volcano plots representing significant up- (red dots) and down-regulated (blue dots) genes of treated *E. coli* strains compared to that of C40. (**C) (a**) GO classification of DEGs based on the biological processes of GO terms in percentage of detected significant DEGs; (**b**) number of DEGs selected according to phenotype expression in various treatments. (**D)** Heatmap of DEGs classified based on phenotype traits related to antibiotic resistance, response to heat and cold, regulation of cell shape and growth, biofilm formation, and flagellum-related genes.

The FastQC was used for validation of the trimmed reads and gene expression level was calculated for all tested strains. By this analysis, 4,400 transcripts were identified in all strains. From reads per kilobase per million (RPKM) values of these transcripts, differential expression analyses were performed in comparison to that of C40. In total, 4,079 transcripts were obtained for ZnO40, 4,090 for ZnONPs40, 4,062 for ZnO20+20, and 4,099 for ZnONPs20+20. Principal component analysis was performed from RPKM values of all transcripts (Fig. 3A). The Pearson correlation test supported results from PCA (Table S3A and C). Treatments were divided into five clusters, with the most similarity between treatments C40 and ZnO20+20 (88%). The second highest similarity was seen between ZnO40 and ZnONPs40 (84%). Volcano plots (Fig. 3B) were constructed to separate the differentially expressed genes (DEGs) (*P* ≥ 0.05, log2FC ≥ ±1.5) calculated as the ratio of Zn-treated bacteria (ZnO40, ZnONPs40, ZnO20+20, ZnONPs20+20) to C40. Numbers of up-regulated and down-regulated genes are shown in Fig. 3B. The most up-regulated genes were found in *E. coli* under ZnO40 treatment. In contrast, ZnONPs40 and ZnO20+20 had the lowest number of down-regulated genes. Under ZnONPs20+20 treatment, the number of up-regulated genes was low, indicating great down-regulation. Overview of numbers and percentage representation of transcripts are shown in Table S4A.

Changes in biological processes based on the gene ontology (GO) in all Zn treatments were detected in comparison to that of C40 and are visualized in Fig. 3Ca as a percentage to all significant DEGs. In all treatments, the most affected were the metabolic processes, followed by the cellular processes and responses to stimulus. For the further analyses, only significant DEGs relevant to the tested phenotype including antibiotic resistance, response to heat, bacterial growth, cell shape, biofilm formation, flagellar system, and genes related to zinc were selected. The numbers of selected genes were assigned to GO parent classes (Fig. 3Cb).

According to the parent classes of GO biological processes, relevant DEGs corresponding to the phenotype were classified based on eight groups of genes: response to antibiotics and xenobiotics and transport activity, heat, cold, regulation of cell shape and cell growth, biofilm formation and quorum sensing, flagellum-related genes, and zinc-related genes. The total numbers of genes selected based on phenotypic expression were 44, 14, 10, and 1 DEGs with up-regulation and 0, 2, 2, and 32 DEGs with down-regulation in ZnO40, ZnONPs40, ZnO20+20, and ZnONPs20+20, respectively. Differential expression of selected genes is demonstrated on a heatmap (Fig. 3D). To further investigate interactions among selected genes, the protein-protein interaction (PPI) network was constructed from the STRING database (Fig. S2A through D). For ZnO40 treatment, selected DEGs associated with virulence and antibiotic resistance had the highest interaction and the high confidence level, especially for DEGs related to transcription and translation. The number of interactions among DEGs in bacteria from other treatments was insufficient, with exception of ZnONPs20+20. Transcriptome data were validated by the real-time quantitative polymerase chain reaction (qPCR) for selected DEGs (*yiaG*, *sodC*, *osmY*, *otsB*, and *rpsL*) (Fig. S3).

### Proteomic analysis

**Fig 4:**
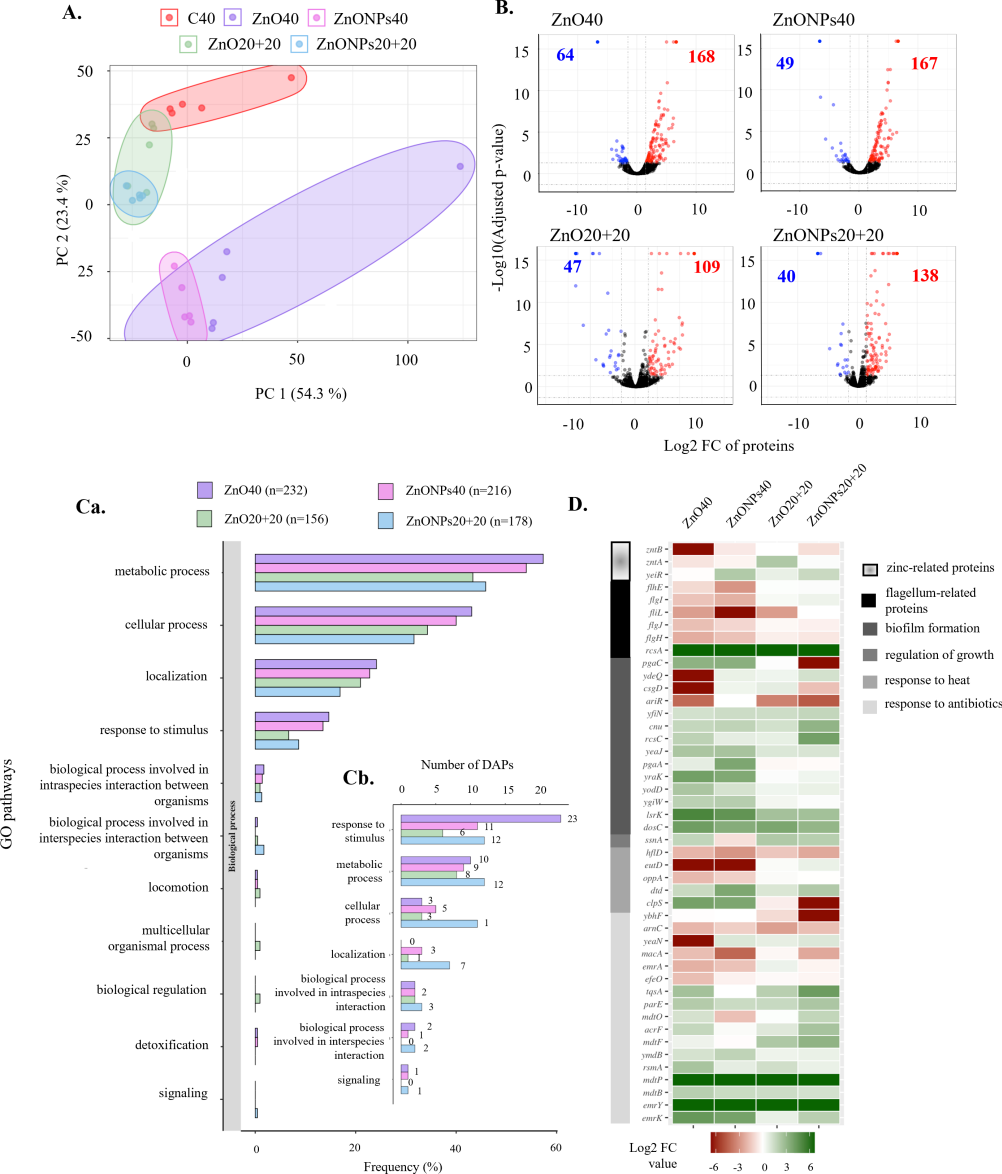
Proteomic analysis. (**A)** PCA plots of biological pentaplicates for various treatments; (**B)** volcano plots representing up- (red dots) and down-regulated (blue dots) genes of treated *E. coli* compared to that of control C40; (**C) (a**) GO classification of DAPs based on the biological processes of GO terms in percentage of detected significant DAPs; (**b**) a number of DAPs selected based on phenotype expression in various treatments; (**D)** heatmap of DAPs classified according to phenotype traits related to antibiotic resistance, response to heat, regulation of cell shape and growth, biofilm formation, and flagellum-related proteins.

Total number of detected proteins with at least two unique peptides and with non-zero abundance in at least three out of five replicates from ZnO40, ZnONPs40, ZnO20+20, and ZnONPs20+20 treatments was 2,494, 2,493, 2,497, and 2,484, respectively. Principal component analysis was performed from protein abundances of all proteins (Fig. 4A). The Pearson correlation test supported results from PCA (Table S3B and D). Bacterial treatments were divided into five clusters with the most similarity between C40 and ZnO20+20 (98%) and C40 and ZnONPs20+20 (97%). In contrast, extended bacterial treatments (ZnO40 and ZnONPs40) compared to that of C40 resulted in lower similarity (85%). Volcano plots (Fig. 4B) were constructed under the same conditions as in the analysis of transcriptomic data. The most up- and down-regulated proteins were found in *E. coli* under the ZnO40 treatment, followed by ZnONPs40, ZnO20+20, and ZnONPs20+20. In contrast, ZnONPs40 and ZnO20+20 resulted in the lowest number of down-regulated genes (Fig. 4B). Overview of numbers and percentage representation of proteins is shown in Table S4B.

The GO classification of proteomic data was processed the same way as that of transcriptome. Differentially abundant proteins (DAPs) corresponding to the phenotype were categorized to GO terms (Fig. 4Ca). The top GO terms matched the transcriptomic results. The only difference was found in an increased number of proteins across all treatments in localization group over the response to stimulus group. Other GO terms occurred equally to the transcriptomic data with exception of the development process, reproductive processes, and viral processes classes, from which no significant DAPs were identified. As in the DEGs, the DAPs were divided into classes, which were designed according to the observed phenotype characteristics. The total number of proteins selected based on the phenotype were 12, 15, 8, and 17 DAPs with up-regulation, and 12, 6, 3, and 6 DAPs with down-regulation in treatments ZnO40, ZnONPs40, ZnO20+20, and ZnONPs20+20, respectively. Differential abundance of selected proteins in individual groups is demonstrated in the heatmap (Fig. 4D). It represents the same groups as in the transcriptome except for the regulation of cell shape and response to cold. To further investigate interactions among selected proteins, the PPI network was constructed from the STRING database (Fig. S2E through H). For the selected DAPs, the interaction with the high confidence level was observed mainly for ZnO40 treatment but not as much as for transcriptomic data.

### Integration of transcriptomic and proteomic data

**Fig 5:**
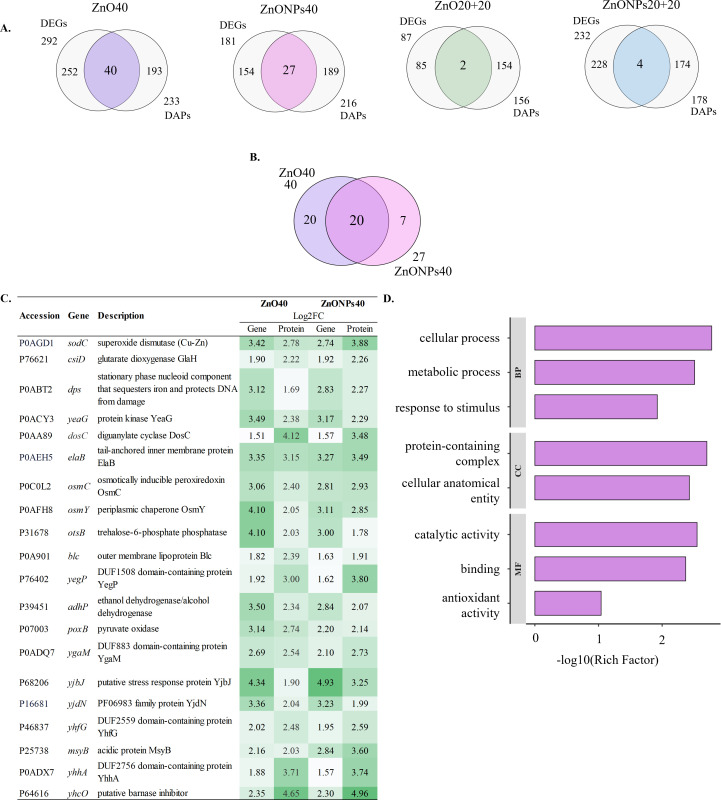
Integration of transcriptomic and proteomic data. (**A)** Venn diagrams of significant DEGs and DAPs for treatments: ZnO40, ZnONPs40, ZnO20+20, ZnONPs20+20. (**B)** Venn diagram indicating common significant DEGs and DAPs of ZnO40 and ZnONPs40 treatments. (**C)** Table of common significant DEGs and DAPs of ZnO40 and ZnONPs40 treatments with log2FC values. (**D)** GO enrichment analysis for only common transcripts and proteins in ZnO40 and ZnONPs40 treatments for BP, CC, and MFs.

Overlaps between DEGs and DAPs are shown in Fig. 5A. The highest number of co-altered genes/proteins was found in ZnO40 (*n* = 40) and ZnONPs40 (*n* = 27) treatments, while ZnO20+20 and ZnONPs20+20 treatments shared only two and four genes/proteins, respectively. Overlaps were compared among all treatments, but only ZnO40 and ZnONPs40 treatments shared 20 significantly (*P*-value ≤ 0.05, log2FC value ±1.5) co-affected genes/proteins (Fig. 5B) with at least threefold increase in expression/abundance (Fig. 5C). The GO enrichment analysis revealed involvement of DEGs/DAPs in cellular, metabolic processes, response to stimulus in biological process (BP), in catalytic activity, binding and antioxidant activity in molecular function (MF) and in protein-containing complex, and cellular anatomical entities in cellular complex (CC) (Fig. 5D). STRING analysis of these common DEGs/DAPs revealed their interaction and production in response to stress (AdhP, Blc, CsiD, Dps, ElaB, OsmC, OsmY, OtsB, SodC, YeaG, YegP), and with a role in metal ion binding (AdhP, DosC, Dps, OtsB, PoxB, SodC, CsiD). In addition, AdhP and SodC are associated with zinc binding (Fig. S4).

## DISCUSSION

Zinc is an essential trace element for all organisms, including bacteria; however, an extended exposure to Zn can promote commensal-to-pathogen transition (15) and biofilm formation, and increase intercellular interactions (31). The specific mechanisms and characteristics of these effects are currently unknown. Bacteria can adapt to changes in their environment due to their genetic flexibility (32, 33). As an example, resistance to zinc and antibiotics was observed in intestinal *E. coli* from livestock (34). Our previous study demonstrated a higher frequency of virulence factors in *E. coli* from piglets fed zinc-supplemented diet (35). In the current study, we aimed to investigate reversible/irreversible changes caused by zinc exposure in *E. coli* on phenotypic, genomic, transcriptomic, and proteomic levels with the focus on antibiotic resistance and virulence.

### Extended ZnO/ZnONPs exposure increased antibiotic resistance

Previous studies showed a link between zinc and the evolution of bacterial antibiotic resistance (36). In our study, an extended exposure to ZnO led to the increased MIC of aminoglycosides (GEN, AMK, NTL, TOB), cephalosporins (CFZ, CTZ), and sulfonamides (SXT), indicating potential development of resistance. According to the EUCAST, the critical breakpoint limit was reached for GEN, AMK, and CFZ (30). In the treatment with ZnO stopped at 20th sub-culture, the MIC values returned to the original levels, with the exception of AMK.

Aminoglycoside resistance mechanisms include mutations in genes coding for 16S rRNA, production of ribosome-modifying enzymes (methyltransferases), AG-modifying enzymes, and efflux pumps (37, 38). Mutations in genes encoding 16S rRNA and in case of GEN, also in 23S rRNA, result in aminoglycoside resistance (39–41). However, these mutations were not observed in our study. Therefore, the resistance to aminoglycosides could be caused by: (i) an inactivation of the electron transfer chain when the bacteria switch from aerobic to anaerobic respiration state making them less susceptible to aminoglycosides (42); (ii) removal of aminoglycosides from the cells by efflux systems and; (c) increased production of ribosomal proteins. The transcriptomic analysis revealed an increased production of small ribosomal subunits in the ZnO40 treatment which might lead to aminoglycoside resistance.

Resistance mechanisms to cephalosporins include the production of the intrinsic β-lactamase AmpC (43), acquired extended spectrum β-lactamases (ESBLs) TEM, SHV, CTX-M and their mutated variants, and the production of efflux pumps (44). Increased production of AmpC was not observed in our treatments; however, all treatments resulted in the accumulation of RcsA that is linked to the response to ampicillin (45). Therefore, this protein indicates a risk of developing resistance to ampicillin and other β-lactams. Although this resistance was not phenotypically detected in our study with the exception of resistance to cephalosporins.

Proteomic analysis revealed an altered production of the efflux pump components of the major facilitator superfamily (MFS), ATP-binding cassette (ABC), resistance-nodulation-division transporter superfamily, and exporters regulating directed efflux of antibiotics out of the cell to confer antibiotic resistance (EmrKY, EmrA, MacA, MdtB, MdtF, MdtP, AcrF, ArnC, ZntB) with the least affection from ZnONPs20+20 treatment. The accumulation of EmrKY, as part of the EmrKY-TolC efflux pump from the MFS component, in ZnO40 likely conferred phenotypic antibiotic resistance (46, 47). The abundance of EmrK was also elevated in ZnONPs40. However, an elevated production of efflux pumps components linked to antibiotic resistance (EmrY and MdtP) was also detected in treatments (ZnONPs40, ZnO20+20, ZnONPs20+20) without the phenotypic expression. Recently, it was found that persistent bacteria continuously express defense mechanisms (e.g., efflux pumps), in response to adverse conditions, such as antibiotic exposure (48, 49). Our observation of the increased abundance of EmrY and MdtP in all treatments indicated that both forms of zinc may induce a higher persistence. After the withdrawal of ZnO in ZnO20+20 treatment, bacteria continued manifesting phenotypic resistance to AMK. This could be due to the production of the same efflux pumps as those in the ZnO40 treatment. The reversal of the phenotypic expression to other antibiotics may be due to the stabilization of the efflux pump and the respiratory system. It is known that in bacteria, Zn2+ homeostasis is maintained by the Zn2+ uptake/efflux systems (43). These two systems provide a balance between the requirement for this element and its toxicity, and therefore, it is essential for bacterial growth and survival (50).

The phenotypic data have confirmed an increased resistance to SXT in cells under the ZnO40 treatment. Mechanism of resistance to sulfonamides and trimethoprim is due to a permeability barrier, a naturally insensitive intrinsic dihydrofolate reductases (DHFR), and spontaneous chromosomal mutations in the intrinsic DHFR (*folA*) gene involved in the folic acid pathway (51). In our study, we detected the increase of transcriptional regulation of the *folA* gene in ZnO40 and ZnONPs40 treatments and this likely resulted in SXT resistance (52).

The extended treatment of *E. coli* with ZnONPs (ZnONPs40) led to a lower sensitivity to CHL only. The most common resistance mechanisms to CHL include the production of acetyltransferases, target site mutation/modification, decreased outer membrane permeability, and efflux pumps that often act as multi-drug efflux transporters (53–55). Overall, our data indicate that the use of ZnONPs does not lead to antibiotic resistance development, and it may even slow it down. However, an increase of efflux pumps abundance on the proteomic level in bacteria exposed to ZnONPs suggests that antibiotic resistance cannot be ruled out. In addition, the extended exposure of sub-lethal ZnONPs may increase the rate of horizontal gene transfer of antibiotic resistance traits (56).

### Extended ZnO/ZnONPs treatments affected cell morphology

Scanning electron microscopy revealed changes in the shape and size of bacterial cells exposed to ZnO40 and ZnONPs40. Bacterial shape affects critical biological functions, including nutrient acquisition, motility, dispersal, stress resistance, and interactions with other organisms. Critical roles of morphogenesis in bacteria-host interactions, including biofilm formation and pathogenesis, have been demonstrated previously (57, 58). The increase of the cell thickness and the change from a rod-like to a more coccoid shape is likely related to changes in proteins involved in the flagellar rod formation. The increased expression of the *ldtD* gene coding the periplasmic L,D-transpeptidase with a role in the protective re-modeling of peptidoglycan during the cell envelope stress (59) was detected in our transcriptome analysis in ZnO40 treatment. The production of LdtD murein transpeptidase responsible for the strengthening of peptidoglycans, thus making the cell envelope impermeable to toxic compounds and antibiotics, is likely a result of the continuous stress conditions caused by this treatment (60). LdtD overproduction also confers broad-spectrum resistance to β-lactams that is corroborated by resistance to cephalosporines (cefotaxime [CTX]). In a recent study, it was demonstrated that ampicillin causes peptidoglycan polymerization requiring the L,D-transpeptidase activity of LdtD (61). LdtD physically interacts with the penicillin-binding protein PBP when cells are experiencing the outer membrane stress; LdtD acts together with PBP to produce a highly cross-linked nascent peptidoglycan in *vitro* (59). Peptidoglycans in bacteria exposed to ZnO and ZnONPs were likely strengthened by the production of LdtE. Expression of *ldtD* and *ldtE* is regulated by the RNA polymerase sigma S factor (RpoS). This factor encoded by *rpoS* induces the general stress response and regulates expression of a wide variety of genes (62). The *rpoS* in C40, ZnO20+20, and ZnONPs20+20 had the stop codon but not in ZnO40 and ZnONPs40. As the shape and size of cells in the ZnO40 and ZnONPs40 changed, it indicates that the gene produced a functioning protein in these cells, resulting in the differential expression of *ldtD* and *ldtE. Escherichia coli* remodels and recycles nearly a half of its existing peptidoglycan during the cell division, and its catabolism is driven by the murein-degrading enzymes (63). In *E. coli*, the protein MltF (63) is involved in this process and it is also responsible for cell-wall excavation to enable the passage of pili, fimbriae, and other macromolecular complexes through the peptidoglycan (64). This gene had an SNP mutation in ZnO40 treatment. Possible inactivation of this gene could result in the altered phenotype and consequently to LdtD protein overproduction, potentially leading to antibiotic resistance.

### Extended ZnO/ZnONPs treatments altered bacterial virulence

We found that in bacteria with extended exposure to ZnO and ZnONPs (ZnO40, ZnONPs40), the production of key virulence factors involved in quorum sensing, motility (pili, flagella), and biofilm formation was significantly affected. Many studies have addressed the question on whether pollutants influence bacterial virulence (15, 31, 50). One of the goals of our study was to determine whether zinc in forms of ZnO or ZnONPs have any effect on the ability of bacteria to produce adhesins, biofilm-related proteins, and on quorum sensing. Phenotypic analysis revealed a greater biofilm formation in ZnONPs20+20 and ZnO40 treatments and a lower biofilm formation in the ZnONPs40 treatment when compared to that of C40. ZnO and ZnONPs treatments altered the production of biofilm-related proteins (adhesins, autoinducers, regulators, or repressors of biofilm formation) and thus potentially increased bacterial virulence.

Biofilm is formed by adaptation of bacterial cells to changing environmental conditions and requires coordinated regulation of a large set of genes. There are 16 regulators controlling several hundred genes important for the initiation of biofilm formation (65). Overproduction of DosC and LsrK in all bacteria exposed to zinc in our study could have a deleterious effect on cell division and might increase and regulate biofilm formation via the quorum sensing system and decrease motility (66–68). Moreover, elevation of DosC in ZnO40 and ZnONPs40 treatments was detected at the transcriptional and translational level. At the same time, this could lead to lower motility and stimulation of the expression of *csgBAC* and *pgaABCD* involved in biofilm formation processes (67, 69). Higher accumulation of proteins YgiW, YmdB, YodD, YraK, and PgaA related to biofilm formation (70) was observed in bacteria under extended Zn treatments (ZnO40, ZnONPs40).

The type III secretion system (T3SS) and the bacterial flagellum are pathogenicity-associated structures found at the surface of many disease-causing bacteria. The decreased production of FlgJ, FlgI, FliL, and FlhE of the flagellar-specific T3SS utilizing an injectisome T3SS to elicit cytotoxicity toward epithelial cells and macrophages (71) was detected in bacteria exposed to ZnO40, ZnO20+20, and ZnONPs40. The lower abundance of the flagellum-proteins leads to lower motility and results in a tendency of bacteria to form the biofilm (72). The T3SS system ensures the transport of effector proteins playing a role in quorum sensing and thus in biofilm formation (73). In addition, genome sequencing revealed the deletion of hypothetical proteins and T6SS PAAR proteins in bacteria exposed to ZnONPs40. This deletion could explain the formation of weaker biofilm under this treatment in comparison to that of C40 treatment. On the other hand, the increased biofilm production in ZnONPs20+20 treatment could be explained by regaining regular bacterial fitness but with modifications after the treatment end. However, this remains to be investigated further.

Overall, the effect of extended exposure of bacteria to ZnO/ZnONPs on biofilm formation is unclear and requires further studies. Nevertheless, zinc significantly altered the expression of genes and the production of proteins involved in biofilm formation.

### Extended ZnO/ZnONPs treatments altered bacterial growth

For all treatments, as the temperature increased, the growth rate of *E. coli* decreased. The growth rate and growth intensity were most negatively affected in bacteria under extended ZnO and ZnONPs treatments (ZnO40 and ZnONPs40). In response to heat, these treatments resulted in production of more proteins necessary for the quality control of proteins. High levels of heat shock proteins are produced in response to free oxygen radicals generated in bacteria under stress conditions (74) and these were likely generated under ZnO and ZnONPs treatments (75). The growth in bacteria under ZnO40 and ZnONPs40 exposure was likely influenced by increased expression of the gene encoding the acyl carrier protein AcpP which regulates the metabolism of unsaturated fatty acids (76).

Reduced growth of ZnO40 and ZnONPs40 treatments in temperatures above 37°C is likely a reflection of greater accumulation of the ClpS. By assisting in the proteolysis, disaggregation, and refolding of the aggregated proteins, caseinolytic proteases (ClpS) enhance the cellular survival under stress conditions (77, 78). A higher abundance of ClpS was also detected in bacteria exposed to ZnONPs20+20, suggesting that the persistence of the greater cell survival under adverse conditions was supported by ZnONPs even after its withdrawal.

The increased expression of genes in the class of homoserine lactones (*hslJ, hslO, hslR, hslU,* and *hslV*) was found in the ZnO40 treatment, while ZnONPs40 had higher expression of only *hslR* that encodes Hsp15. In our study, *E. coli* was cultured at 37°C and we hypothesize that the heat shock was substituted by the extended zinc pressure and resulted in an activation of the rescue mechanisms including the higher production of Hsp15 (79–83). The heat shock protein Hsp15 is involved in ribosome recycling and *hsl* genes expression is mainly related to a heat shock. The up-regulated *hslO* gene in ZnO40 encodes the zinc-dependent, redox-regulated chaperone Hsp33 responsible for extending the lifespan of bacteria during the heat stress (84). The induction of heat shock proteins (Hsps) is considered as an important protective, physiologically adaptive, and genetically conserved response to the environmental stress in all organisms. As demonstrated in the previous study on *Tigriopus japonicus*, putative xenobiotic elements indicated that Hsps regulation was caused not only by temperature but also by xenobiotics (83). Our data showed that the extended Zn exposure also resulted in the up-regulation of these genes although the significance of this for the bacterial cells remains to be investigated.

### Extended ZnO/ZnONPs treatments influenced bacterial defense against ROS

ZnO40 and ZnONPs40 treatments had an overlap of 20 significantly co-altered DEGs/DAPs from which 11 were linked to stress (AdhP, Blc, CsiD, Dps, ElaB, OsmC, OsmY, OtsB, SodC, YeaG, YegP) and seven were associated with metal binding (AdhP, DosC, Dps, OtsB, PoxB, SodC, CsiD). Overproduction of ROS can damage the bacterial DNA and induce resistance under the antibacterial pressure. However, bacteria are also able to activate antioxidant systems to eliminate intracellular ROS (85). This can enhance transcription of genes related to antioxidant systems such as *sodC* (62), which was shown in our study in ZnO40 and ZnONPs40 treatments. Oxidative stress can be also stimulated by starvation. An increased expression/abundance of Dps, known as a DNA “protector during starvation” (86), leads to protection of the cell from multiple stresses including oxidative stress and can contribute to tolerance to chemicals. Dps binds tightly to DNA and forms a DNA-protein crystal that protects the DNA from damage (86). Expression of CsiD is induced by carbon starvation and leads to increased tolerance to furfural (87, 88). Dps and CsiD play a role in collecting iron, which serves as a reducing agent in oxidative damage protection/repair (87, 89). According to a recent study (90), iron stress increased the production of YeaG, which plays a role in adaptation to chronic nitrogen deficiency and participates in protein phosphorylation (91). Overexpression and abundance of DosC, ElaB, OsmC, OsmY, and OtsB are signs of cell reaction to the stimulus causing stress and cellular imbalance. These proteins are involved in many cellular pathways participating in response to osmotic pressure, peroxidase, hydrolase activity, chaperone-mediated protein folding (OsmC, OtsB, OsmY), and cellular virulence as biofilm-associated DosC increases biofilm formation and decreases motility as mentioned above. The overproduction of OsmC, OsmY, and OtsB is a response to osmotic stress that may be caused by release of Zn2+ from both zinc forms. Likewise, an excess of the inner membrane and ribosome binding protein ElaB together with YgaM increases cell survival under the heat and oxidative stress and leads to inhibition of cell growth (92). Increased expression and abundance of enzymes, polypeptides, and proteins associated with response to stress, osmotic influence, hyperoxide, and starvation indicate the cell need to remove or metabolize radicals generated as by exposure to both zinc forms. Due to defense mechanisms against ROS, the cell invests energy in these processes, which may explain retarted cell growth after extended ZnO/ZnONPs exposure (93). Overlapping networks of regulation are common in bacteria and indirect effects of stress responses can also influence virulence factors (94).

### Conclusions

Our study demonstrates that the long-term exposure of *E. coli* to zinc disrupts many physiological processes of the cell, including detoxification activity, response to oxidative stress, and basal metabolism. The phenotypic and multi-omics data revealed the major changes in a cell, including shape, size, growth, and antibiotic resistance and potentially virulence after an extended zinc treatment. The majority of phenotypic alterations were reversed by the termination of ZnO and ZnONPs exposure. These findings indicate that the use of zinc in the form of ZnO and ZnONPs, as feed supplements for livestock, poses a risk to animal and consequently human health.

## MATERIALS AND METHODS

### Effect of an extended exposure of *E. coli* to sub-inhibitory concentrations of ZnO and ZnONPs on minimum inhibitory concentration

*Escherichia coli* ATCC 25922 was grown overnight on 5% Columbia blood agar (Lab Media Servis, Czech Republic) at 37°C. Overnight cultures were diluted in double-concentrated Mueller-Hinton (MH) broth (Sigma Aldrich, USA) to optical density at 600 nm (OD_600_) 0.08–0.13 AU and then diluted 100×. The bacterial solution was aliquoted in 96-well plates containing ZnO/ZnONPs (Sigma-Aldrich, USA) in range 0.125 mg/mL–16.0 mg/mL dissolved in deionized water supplemented with 0.05% (vol/vol) DMSO and cultured overnight (37°C). The following day, the absorbance (620 nm) was measured using the MultiScan EX Microplate Photometer (Thermo Fisher, Germany) and the minimum inhibitory concentration and sub-inhibitory concentration were determined. A loopful (10 µL) of cells from the well with the first concentration below MIC (our sub-inhibitory concentration) were transferred on the surface of 5% Columbia blood agar, spread by streak plate method and cultivated overnight at 37°C. Then, the place with multiple colonies from the agar was harvested by loop and diluted as described above. The whole process was repeated 19 times to get 20 sub-cultures in seven independent replicates with MIC measurement. In parallel, mock-treated bacteria were incubated under the same conditions using PBS (control) (95). This was performed for the control treatment (C40) through the whole sub-culturing as well. After reaching the 20th sub-culture, the treated bacteria were subdivided into two lines and the experiment continued for additional 20 cycles with ZnO/ZnONPs (ZnO40, ZnONPs40) or mock (ZnO20+20, ZnONPs20+20). Every bacterial sub-culture was stored in cryo-medium (−80°C) until further use. Used ZnONPs had hexagonal shape as previously characterized by Paszek et al. (96) and Daoud et al. (97). Considering the same molecular weight and the concentration of ZnO and ZnONPs, it is expected that the range of total dissolved zinc concentrations are comparable.

### Antibiotic susceptibility

Heptaplicates *of E. coli* 40th sub-cultures (C40, ZnO40, ZnO20+20, ZnONPs40, ZnONPs20+20) grown overnight (37°C) on 5% Columbia blood agar were exposed to antibiotics commonly used in the treatment of Gram-negative bacteria using MICRO-LA-TEST (Erba-Lachema, Germany). Chosen antibiotics for MIC determination were ampicillin, ampicillin/sulbactam, piperacillin, piperacillin/tazobactam, aztreonam, CFZ, cefuroxin, CTX, CTZ, cefoperazone, cefoperazone/sulbactam, cefepime, GEN, AMK, NTL, TOB, SXT, ciprofloxacin, meropenem, ertapenem, tigecycline, tetracycline, CHL, and colistin. For this analysis, 96-well plates (G^-^I and G^-^II) containing prepared antibiotics were used according to the manufacturer’s instructions. Minimum inhibitory concentrations of antibiotics were evaluated after overnight growth. One replicate, whose value differed from the others, was excluded from final evaluation.

### Cell morphology and thickness

Grown *E. coli* colonies were diluted in 2× MH broth and cultivated at 37°C overnight. Then, the strains were centrifuged (1,000 × *g*, 5 min) and 1 mL of phosphate buffer saline (PBS) was added to obtain bacterial pellet. It was incubated at 37°C, 45 min, 40 × *g*. The bacteria were centrifuged (3,000 × *g*, 2 min) and obtained pellet was washed three times with PBS. The pellet was incubated with glutaraldehyde (1%) for 30 min in the dark at room temperature. Bacteria were washed three times with MilliQ water. Then 1 mL of MilliQ water was added to the strains, incubated for 10 min and centrifuged (3,000 × *g*, 2 min). The washed bacteria were dehydrated in several steps with an ascending ethanol series ranging from 40% to 100%. Each time, the appropriate percentage of ethanol was added to the strains, which were then incubated for 5 min before being centrifuged (3,000 × *g*, 2 min). Bacteria with 100% ethanol were incubated for 15 min, washed with twice with 100% ethanol and centrifuged (3,000 × *g*, 2 min). Scanning electron microscopy was used to examine the morphology on a Tescan MAIA 3 equipped with a field emission gun (Tescan Ltd., Brno, Czech Republic). The most promising images were obtained using the In-Beam SE detector at a working distance was approximately 3.00 mm and at 2 kV acceleration voltages. 768 × 858 pixels images were obtained at 22,100-fold magnification covering sample area of 9.392 µm^2^. Full frame capture was performed in ultra high resolution mode and accumulation of image with image shift correction enabled, and it took about 0.5 min with the 1 µs/pixel dwell time. The spot size was set at 4.14 nm. Analysis was performed on seven randomly chosen cells of the picture for each sample and visualized by bar graph and statistically evaluated by Welch’s two-sample unpaired *t*-test in GraphPad Prism 8.

### Biofilm formation

Overnight bacterial cultures were diluted in tryptic soy broth (TSB, Oxoid, Hampshire, UK) containing 1% of glucose to obtain absorbance equal to OD_600_ = 0.1 AU. Two hundred microliters of each culture solution was pipetted into wells of the plate for 7 days of incubation with daily refreshing of the TSB with 1% glucose. After incubation, the formed bacterial biofilm was washed three times with PBS. The viability of the biofilm was determined by the addition of Alamar Blue with PBS (ratio 1:9) (Thermo Fisher Scientific, Waltham, MA, USA), the fluorescence of each well was evaluated (560/590 nm, excitation/ emission) and all the strains were compared to control strain C40. The analysis was performed in 30 repetitions for each sample and visualized by bar graph and statistically evaluated by Welch’s two-sample unpaired *t*-test in GraphPad Prism 8.

### Bacterial growth

The treatments of *E. coli* ATCC 25922 (C40, ZnO40, ZnONPs40, ZnO20+20, ZnONPs20+20) were cultivated at 37°C overnight on 5% Columbia blood agar plates. Bacteria were diluted in MH broth (Oxoid, Hampshire, UK), where their concentration was determined by measuring OD_600_ to 0.1 AU. The absorption intensity of bacterial samples corresponds to ∼1.5 × 108 CFU/mL (where CFU is colony forming unit). Finally, the bacterial cultures were further diluted 100× in MH broth. One hundred microliters (∼1 × 10^6^ CFU/mL) of each treatment of *E. coli* was diluted in MH broth and placed into a Honeycomb plate. The bacterial growth was determined using Bioscreen C (Oy Growth Curves Ab Ltd., Helsinki, Finland). The OD_600_ readouts were measured every 30 min for 24 h at 37°C, 41°C, 43°C, and 46°C. After analysis, the growth curves were visualized and the influence of ZnO and ZnONPs on the growth of *E. coli* in different temperatures was assessed.

### Cell preparation for multi-omic analysis

*Escherichia coli* ATCC 25922 40th sub-cultures (C40, ZnO40, ZnONPs40, ZnO20+20, ZnONPs20+20) were spread on tryptone soya agar and cultured for 16 h (37°C). Then, bacteria were diluted in PBS to have 3.6 × 10^9^ CFU/mL and centrifuged (1,500 × *g*, 4°C, 15 min). Supernatant was removed from the bacterial pellet; 10 mL of PBS was added to it and mixed. Then, it was aliquoted into 1.5 mL Eppendorf tubes (1 mL) and centrifuged (1,500 × *g*, 4°C, 15 min). The supernatant was removed again. The bacterial pellets were frozen in liquid nitrogen and put at −80°C until further use. For multi-omic approach, one replicate of each treatment was used for genomic analysis, three replicates of each treatment were used for transcriptomic analysis, and five replicates of each treatment were used for proteomic analysis.

### Genome analysis

Genomic DNA for whole-genome sequencing was extracted using NucleoSpin Tissue kit (Macherey-Nagel, GmbH & Co. KG, Germany) for one replicate of each treatment (*n* = 1). Afterward, the library was prepared by Nextera XT Library Preparation kit (Illumina, San Diego, CA, USA) and sequenced using 2 × 250 bp paired-end sequencing using the NovaSeq 6000 platform (Illumina). Raw reads were trimmed using Trimmomatic v0.39 (98) to remove adapter residues and low quality regions with quality threshold set to 20 (Q ≤ 20). SPAdes v3.13.1 (99) with the “--careful” configuration was used to obtain *de novo* assemblies. ABRicate (100) was used to identify plasmid replicons and resistance genes of the control (C40) and treated bacteria (ZnO20+20, ZnO40, ZnONPs20+20, ZnONPs40) comparing to databases PlasmidFinder and ResFinder (101, 102) with threshold 90% coverage and 90% identity. To characterize mutations created after the zinc treatment, the analysis of genomic variants was performed. First, Bowtie2 v2.3 (103) was used to map quality- and adapter-trimmed reads of treated strains to the *de novo* assembly uninfluenced control *E. coli* ATCC 25922 (C40). Variant calling (single nucleotide polymorphisms, insertions, and deletions) was performed by VarScan v2.4.4 (104) considering the minimum read coverage of 20. Variant frequency threshold was 80% and called variants were manually curated in Geneious v7.1.9 (Biomatters, Auckland, New Zealand).

### Transcriptome analysis

RNA was extracted by TRIzol Reagent according to the manufacturer’s instructions (TRIzol Reagent, Invitrogen, Carlsbad, CA), using zirconium beads (Benchmark Scientific, Sayreville NJ, USA) for homogenization of the bacterial pellet from three replicates of each strain (*n* = 3). The only difference was after homogenization, when the samples were put in the freezer (−20°C) for 10 min. Then, the protocol was followed. Extracted RNA was purified by ethanol precipitation protocol according to manufacturer´s instructions. The concentration of RNA was checked on the NanoDrop OneC Spectrophotometer (Thermo Fischer Scientific, Waltham, MA, USA). The QIAseq Stranded RNA Library Kit (Germantown, MD, USA) in combination with QIAseq UDI Y-Adapter Sequences (Germantown, MD, USA) was used for the preparation, amplification, and purification of cDNA libraries (triplicates) according to the enclosed protocol. The quality and quantity of the prepared libraries were determined using the Agilent High Sensitivity DNA Kit (Agilent, Santa Clara, CA, USA) in Agilent 2100 Bioanalyzer (Agilent Technologies, Praha, Czech Republic). The quantity of the library was also determined by a Modulus Single Tube Multimode Reader (Turner Biosystems, Sunnyvale, CA, USA) using a Quant-iT dsDNA Assay Kit (Thermo Fisher Scientific, Waltham, MA, USA). The sequencing was performed on NovaSeq 6000 (Illumina) with a run conjunction of 2 × 150 bp. Obtained data were checked in FastQC (105). From generated reads, adapter sequences were trimmed by BBDuk and low-quality reads (Q ≤ 28) were removed (106). All treatments were mapped to reference *E. coli* NBRC 3301 (NZ_CP048439.1). Gene expression levels were given as reads per kilobase per million mapped reads and differential expression analysis was performed. Differentially expressed transcripts, whose differential expression adjusted *P*-value >0.05, were excluded from GO enrichment analysis. Differential expression log2ratio was used to evaluate expression, and those with differential expression log2ratios greater than 1.5 and less than −1.5 were statistically significant. For estimating log2 fold change and adjusted *P*-values, three types of software were used: the Geneious Prime 2021.0.1 in cooperation with the DESeq2 package (107, 108) and the EdgeR package (109) to calculate differential expression values.

To verify the transcriptomic data, five selected significant genes (*P*-value ≤0.05) were chosen for real-time qPCR analysis. Firstly, cDNA of all strains was prepared by Transcriptor first strand cDNA synthesis kit for RT-PCR (Roche, Germany, Mannheim) according to manufacturer’s instructions. Five hundred nanograms of RNA was used as template for reverse transcription. Prepared cDNA (20 µL) was diluted in UltraPure Dnase/Rnase-Free Distilled Water to a final volume 200 µL. Then, transcriptional expression of these pumps by real-time qPCR was determined in qTOWER3 system (Analytik Jena, Jena, Germany). Gene *tolC* was used as a reference gene. Primers for selected genes were designed using real-time PCR tool on website Integrated DNA Technologies (www.eu.idtdna.com). All the used primer sequences are listed in Table S1A.

### Proteome analysis

Bacterial pellets, from five replicates of each strain (*n* = 5), were extracted by sonication in tert-butyl methyl ether:methanol mixture (3:1). The resulting protein pellets were solubilized (8 M urea, 10 mM dithiothreitol, 100 mM ammonium bicarbonate), alkylated, and digested with trypsin. Portions of samples corresponding to 5.0 µg of peptide were analyzed by nanoflow reverse-phase liquid chromatography-mass spectrometry using a 15 cm C18 Zorbax column (Agilent Technologies), a Dionex Ultimate 3000 RSLC nano-UPLC system, and the Orbitrap Fusion Lumos Tribrid Mass Spectrometer (Thermo) as described previously (e.g., 10.3390/plants9111563). The measured spectra were recalibrated and searched against the *E. coli* UniProt protein database (83333, strain K12) and common protein contaminants by Proteome Discoverer 2.5 (Thermo Scientific), employing Sequest HT, MS Fragger (10.1038/nmeth.4256). Only proteins with at least two unique peptides were considered for the quantitative analyses. For each point, five biologically distinct samples were analyzed. The quantitative analysis was performed by Minora, employing precursor ion quantification followed by normalization and background-based *t*-test. Protein quantification was evaluated as differential expression analysis with resulting data abundance ratios for each treatment compared to that of C40 (ZnO40/C40, ZnONPs40/C40, ZnO20+20/C40, and ZnONPs20+20/C40) and log2 fold change was calculated with the threshold setting to ±1.5 and the *P*-value threshold 0.05.

### Transcriptomic and proteomic data processing/visualization

For transcriptomic and proteomic data visualization PCA, volcano plots, Venn diagrams and GO enrichment analysis for biological process, cellular component, and molecular function were chosen. For transcriptome, PCA plots were generated from log10 RPKM expression values for each strain (15 strains). For proteome, they were generated from log10 abundance values for each strain (25 strains). Firstly, dimensions were calculated in RStudio and dimension 1 (PC1) and dimension 2 (PC2) values were used for PCA plot generation in RStudio via ggplot2. Pearson correlation test of RPKM/abundance values was performed as well. Volcano plots were generated from −log10(differential expression adjusted *P*-value) and differential expression log2 fold change ratio visualized by ggplot2 in RStudio (110). The significance of the obtained differential expression data were determined using the ±log10 adjusted *P*-value. Significantly regulated genes/proteins were subjected to GO enrichment analysis. GO terms were classified according to EcoCyc database: https://biocyc.org/ECOLI/class-tree?object=Gene-Ontology-Terms. The data were visualized in ggplot2 in RStudio (110).

### PPI network construction

STRING v11 (111) database was used to analyze PPI of the selected DEGs, DAPs and overlapped DEGs and DAPs identified in this study and to construct PPI network. At the medium confidence level, the minimum required interaction score parameters were set. DEGs and DAPs with no interaction were removed from the final figure, except the figure with overlapped DEGs and DAPs.

## Data Availability

Raw data generated from the genome sequencing and RNA-Seq experiment of *E. coli* strains were deposited in the Sequence Read Archive (SRA) of NCBI database under the BioProject PRJNA915319, Multi-omic approach of zinc-treated *E. coli*. The mass spectrometry proteomics data were deposited to the ProteomeXchange Consortium via the PRIDE (112) partner repository with the data set identifier PXD027925.

## SUPPLEMENTAL MATERIAL

Figure S1 msystems.00733-23-s0001.tif Cell width measurements in all strains of *E. coli* ATCC25922.

Figure S2 msystems.00733-23-s0002.tif According to selected DEGs, the highest interactions among selected proteins are seen for strain ZnO40.

Figure S3 msystems.00733-23-s0003.tif Log2 fold change values of treated sample transcripts from transcriptomic data compared to log2 fold change of treated sample transcripts from real-time qPCR analysis normalized to *tolC* gene.

Figure S4 msystems.00733-23-s0004.tif In common DEGs and DAPs for ZnO40 and ZnONPs40 treatments: all of them are up-regulated and show strong interactions among each other except Blc, YhcO, YjdN, and YhhA.

Legends msystems.00733-23-s0005.docx Legends for Figures S1 to S4 and Tables S1 to S4.

Table S1 msystems.00733-23-s0006.docx Primers for genes used for qPCR validation of transcriptomic data and changes in MIC of chosen antibiotics.

Table S2 msystems.00733-23-s0007.docx Identified plasmids and resistance genes in all tested strains and identified intergenic mutation.

Table S3 msystems.00733-23-s0008.docx Heatmaps of Pearson correlation values for each bacterial strain.

Table S4 msystems.00733-23-s0009.docx Overview of numbers and percentage representation of transcripts and proteins with distribution according to significance and up/downregulation.

ASM does not own the copyrights to Supplemental Material that may be linked to, or accessed through, an article. The authors have granted ASM a non-exclusive, world-wide license to publish the Supplemental Material files. Please contact the corresponding author directly for reuse.
