# Supplementary figures and images for: Zinc effects on bacteria: insights from Escherichia coli by multi-omics approach
Source: mSystems. 2023 Oct 31;8(6):e00733-23. doi: 10.1128/msystems.00733-23 (PMC10734530; doi:10.1128/msystems.00733-23)

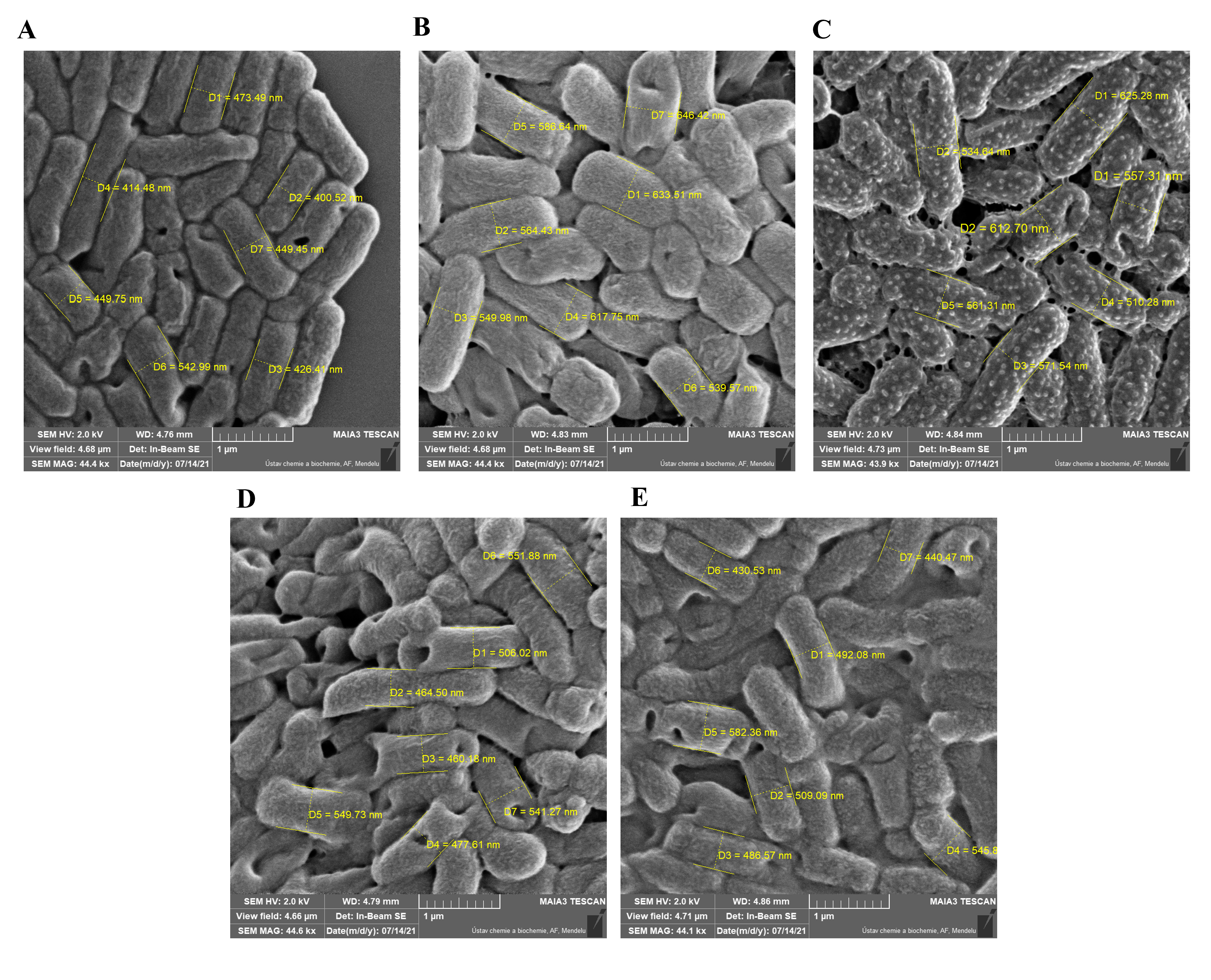

Supplement: Figure S1 — Cell width measurements in all strains of E. coli ATCC25922. [file msystems.00733-23-s0001.tif]

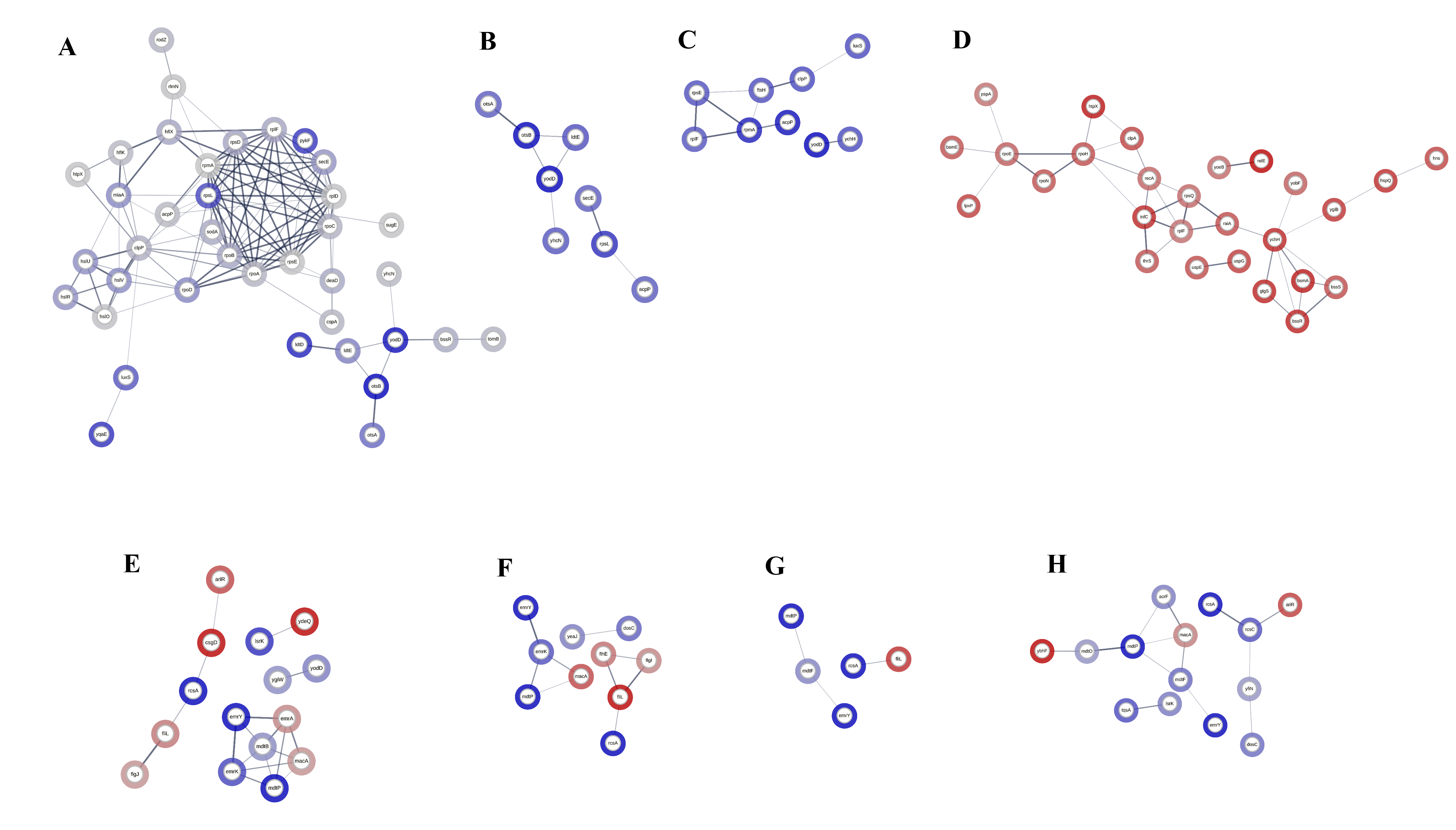

Supplement: Figure S2 — According to selected DEGs, the highest interactions among selected proteins are seen for strain ZnO40. [file msystems.00733-23-s0002.tif]

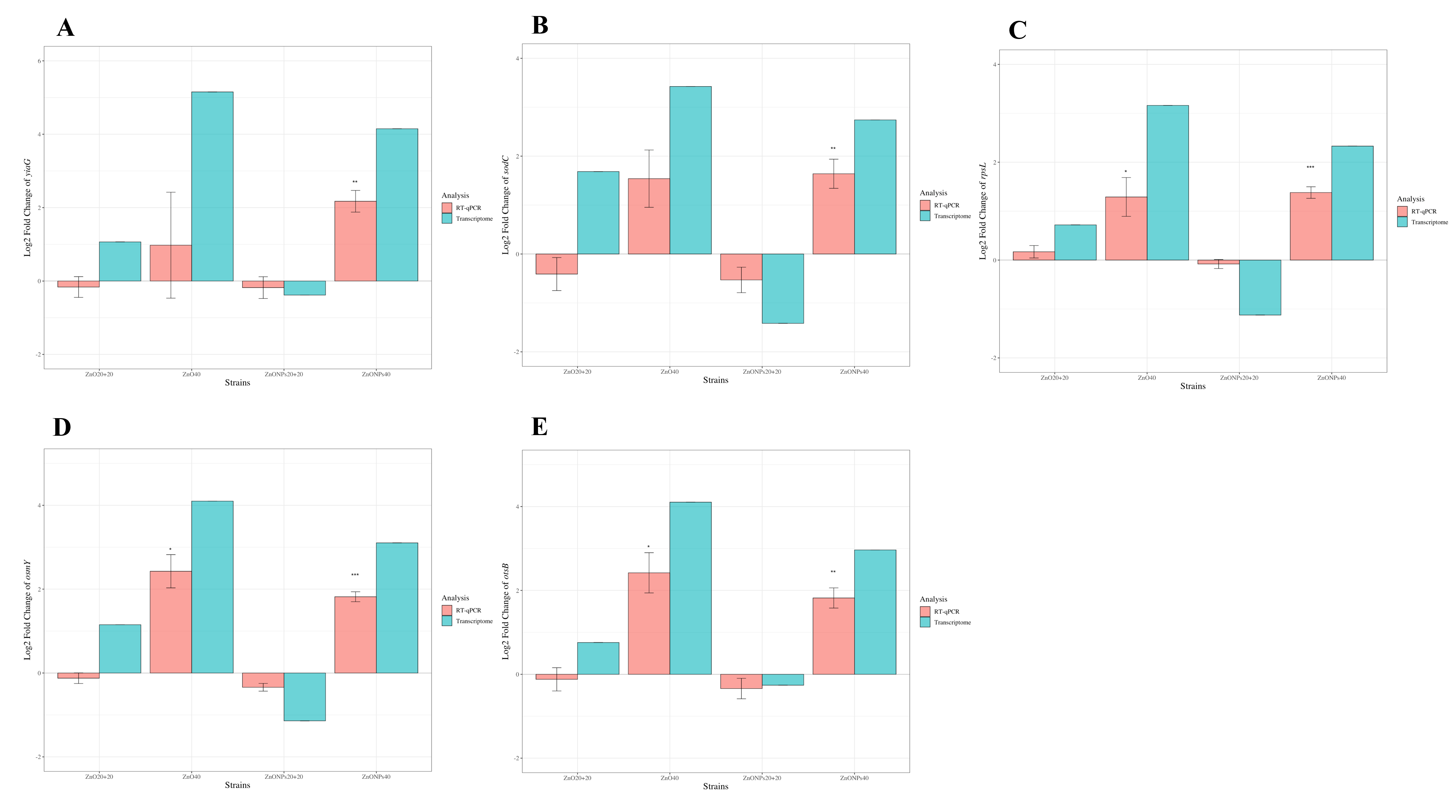

Supplement: Figure S3 — Log2 fold change values of treated sample transcripts from transcriptomic data compared to log2 fold change of treated sample transcripts from real-time qPCR analysis normalized to tolC gene. [file msystems.00733-23-s0003.tif]

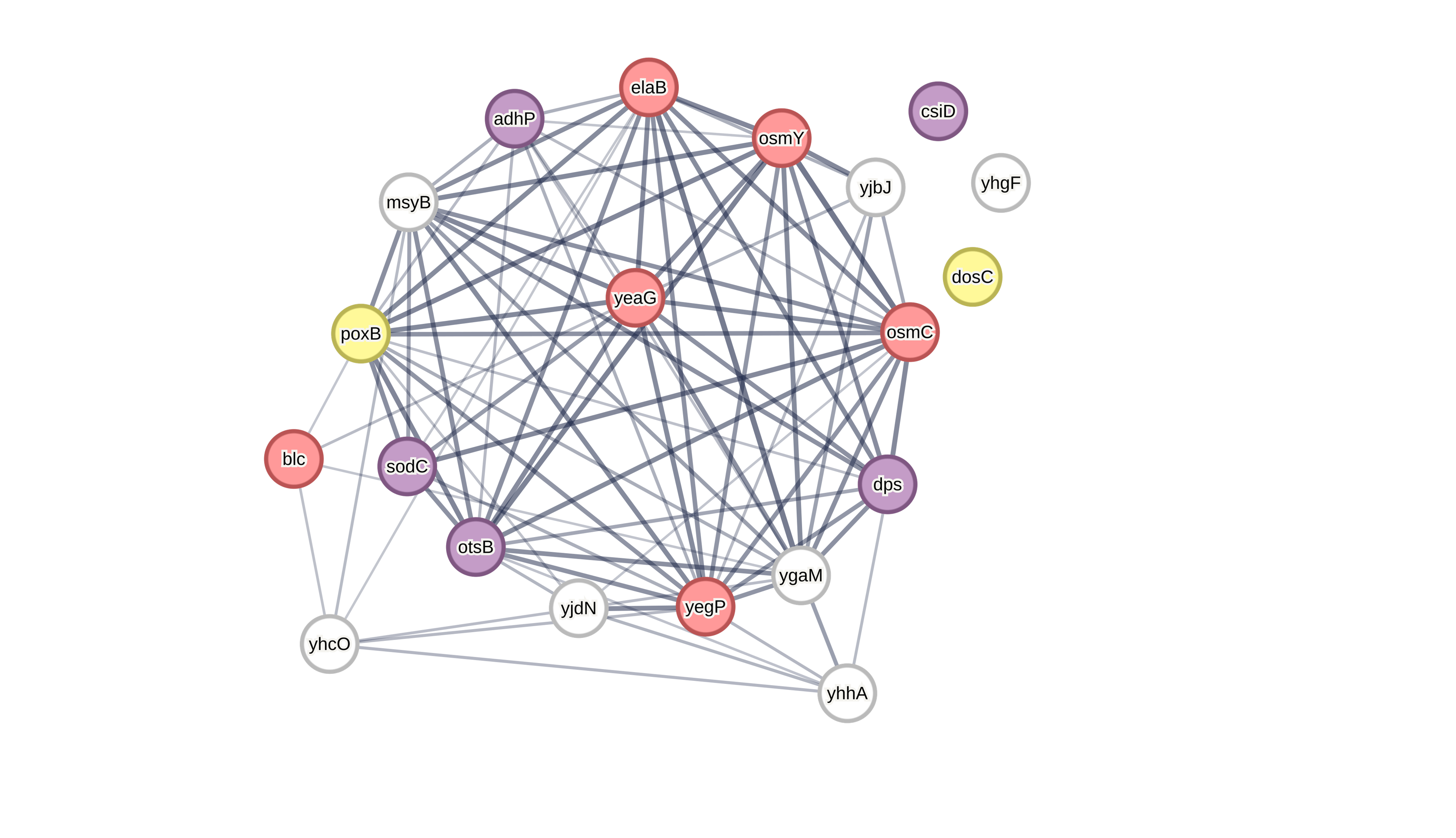

Supplement: Figure S4 — In common DEGs and DAPs for ZnO40 and ZnONPs40 treatments: all of them are up-regulated and show strong interactions among each other except Blc, YhcO, YjdN, and YhhA. [file msystems.00733-23-s0004.tif]
