# Supplementary material for: Zinc effects on bacteria: insights from Escherichia coli by multi-omics approach
Source: mSystems. 2023 Oct 31;8(6):e00733-23. doi: 10.1128/msystems.00733-23 (PMC10734530; doi:10.1128/msystems.00733-23)
Supplement: Legends — for Figures S1 to S4 and Tables S1 to S4. [file msystems.00733-23-s0005.docx]

**Legends of supplementary files**

**Figure S1** Cell width measurements in all strains of *E. coli* ATCC25922: C40 (A), ZnO40 (B), ZnONPs40 (C), ZnO20+20 (D) and ZnONPs20+20 (E) in seven replications.

**Figure S2** According to selected DEGs, the highest interactions among selected proteins are seen for strain ZnO40 (**A**). This strain produces a lot of small (RpsL, RpsE, RpsD) and large (RplF, RplD, and RpmA) ribosomal subunits, as well as RNA polymerase (RpoA, RpoB, RpoC, and RpoD). Small production of ribosomal subunits was observed in strain ZnO20+20 (**C**) (RpsE, RplF and RpmA) and ZnONPs40 (**B**) (RpsL). In both strains, ZnO40 and ZnONPs40, murein synthesis is activated by production of LdtE for ZnONPs40 and by LdtD and LdtE for ZnO40. In ZnO40, the RodZ protein is also produced, affecting the shape of the cell and the proteins synthesized during the temperature shock (HslU, HslV, HslR and HslO). In the ZnONPs20+20 (**D**) strain, all DEGs except KilR are down-regulated. In this strain, there is a decrease in precision during translation (RpsQ) and at the same time a decrease in the production of proteins involved in biofilm repression (BssR, BssS and GlgS). The biofilm is also associated with the strain ZnO40 (BssR) and ZnO20+20 (LuxS and ClpP). Blue halos represents up-regulated transcripts and red halos represents down-regulated halos. Thickess of the lines represents significance of interaction, higher thickess higher interaction. According to selected DAPs, all treated strains produced transporter proteins, with high abundance of pumps associated with resistance to a variety of antibiotics (MdtP, EmrY and other pumps for different strains). In strains ZnO40 (**E**) (EmrK and EmrY), ZnONPs40 (**F**) (EmrK and EmrY) and ZnO20+20 (**G**) (EmrY and MdtF) are produced those that can form complexes and thereby expel antibiotics more effectively. The MacA pump is produced more abundantly in strain ZnO40 than in the other strains (except strain ZnO20+20 with no detection). Flagellar system proteins were produced in all strains (FlgJ,FliL and Flgl are down-regulated except ZnONPs20+20 (**G**) where these proteins were not significantly detected). These proteins are primarily involved in flagellar functionality and flagellar protein transport. Blue halos represents up-regulated transcripts and red halos represents down-regulated halos. Thickness of the lines represents significance of interaction, higher the thickness higher the interaction.

**Figure S3** Log2 Fold Change values of treated sample transcripts from transcriptomic data compared to Log2 Fold Change of treated sample transcripts from real-time qPCR analysis normalized to *tolC* gene. Significance of real-time qPCR samples were determined via calculated SEM values by GraphPad Prism and added to the graphic. Log2 Fold Change values were visualized as histograms for *yiaG* (A), *sodC* (B), *rpsL* (C), *osmY* (D) and *otsB* (E) genes.

**Figure S4** In common DEGs and DAPs for ZnO40 and ZnONPs40 treatments: All of them are up-regulated and show strong interactions among each other except Blc, YhcO, YjdN and YhhA. No interaction among selected DEGs and DAPs are seen for CsiD, YhgF and DosC. Red-colored nodes represent those associated with response to stress [GO:0006950], yellow-colored nodes represent those associated with metal ion binding [GO:0046872], violet-colored nodes represent those associated with both, and white-colored nodes without GO classification.

**Table S1** List of primers for genes used for qPCR validation of transcriptomic data (A). Changes in minimum inhibitory concentration of chosen antibiotics (B): ampicillin (AMP), ampicillin/sulbactam (SAM), piperacillin (PIP), piperacillin/tazobactam (TZP), aztreonam (ATM), cefazolin (CFZ), cefuroxin (CXM), cefotaxime (CTX), ceftazidime (CTZ), cefoperazone (CFP), cefoperazone/sulbactam (SCF), cefepime (FEP), gentamicin (GEN), amikacin (AMK), netilmicin (NTL), tobramycin (TOB), trimethoprim/sulfametoxol (SXT), ciprofloxacin (CIP), meropenem (MEM), ertapenem (ETP), tigecycline (TGC), tetracycline (TCN), chloramphenicol (CHL), and colistin (CST). Dark red color indicates the breakpoint value has been reached, light red color indicates the increasing of MIC value after the treatment in comparison to that of C20 or C40 without breakpoint value reaching. The light green color indicates decreased MIC after treatment in comparison to that of C20 or C40.

**Table S2** List of identified plasmids and resistance genes in all tested strains (C40, ZnO40, ZnONPs40, ZnO20+20, ZnONPs20+20) (A). List of identified intergenic mutations for ZnO40, ZnONPs40, ZnO20+20 and ZnONPs20+20 treatments (B).

**Table S3** Heatmaps of Pearson Correlation Values for each bacterial strain. For transcriptomic data (A), calculated from RPKM values. For proteomic data (B), calculated from protein abundances. Average for each treatment´s Pearson Correlation Values with calculated SEM values are shown for transcriptome (C) and proteome (D).

**Table S4** Overview of numbers and percentage representation of transcripts (A) and proteins (B) with distribution according to significance and up/down-regulation.
