# Supplementary material for: Zinc effects on bacteria: insights from Escherichia coli by multi-omics approach
Source: mSystems. 2023 Oct 31;8(6):e00733-23. doi: 10.1128/msystems.00733-23 (PMC10734530; doi:10.1128/msystems.00733-23)
Supplement: Table S1 — Primers for genes used for qPCR validation of transcriptomic data and changes in MIC of chosen antibiotics. [file msystems.00733-23-s0006.docx]

**Table S1** List of primers for genes used for qPCR validation of transcriptomic data (A). Changes in minimum inhibitory concentration of chosen antibiotics (B): ampicillin (AMP), ampicillin/sulbactam (SAM), piperacillin (PIP), piperacillin/tazobactam (TZP), aztreonam (ATM), cefazolin (CFZ), cefuroxin (CXM), cefotaxime (CTX), ceftazidime (CTZ), cefoperazone (CFP), cefoperazone/sulbactam (SCF), cefepime (FEP), gentamicin (GEN), amikacin (AMK), netilmicin (NTL), tobramycin (TOB), trimethoprim/sulfametoxol (SXT), ciprofloxacin (CIP), meropenem (MEM), ertapenem (ETP), tigecycline (TGC), tetracycline (TCN), chloramphenicol (CHL), and colistin (CST). Dark red color indicates the breakpoint value has been reached, light red color indicates the increasing of MIC value after the treatment in comparison to that of C20 or C40 without breakpoint value reaching. The light green color indicates decreased MIC after treatment in comparison to that of C20 or C40.

| **Primers**  **A** | **Sequences (5´→ 3´)** | **Amplicon size (bp)** | **References** |
| --- | --- | --- | --- |
| Reference gene *tolC* | F: AAGCCGAAAAACGCAACCT | 101 | Swick et al, 2011 |
|  | R: CAGAGTCGGTAAGTGACCATC |  |  |
| *rpsL* | F: GCAAAAACGTGGCGTATGTACTC | 103 | Swick et al, 2011 |
|  | R: TTCGAAACCGTTAGTCAGACGAA |  |  |
| *yiaG* | F: ATTACCCTGACGCACAGAAC | 134 | This study |
|  | R: CGTCTGGATTCCCATTCCTTTA |  |  |
| *osmY* | F: GCTAAAGAAGGCTGGTGAA | 105 |  |
|  | R: TTTCACATGACGGGAAGGG |  |  |
| *otsB* | F: CCGTATTTCTGGGCGATGAT | 94 |  |
|  | R: GTTGCACCTGTGCCAATTT |  |  |
| *sodC* | F: GGTAGCGTCACCATTACTGAA | 105 |  |
|  | R: GCTTCCTTTGGCATGAATATGG |  |  |

| **Groups of ATB**  **B** | **ATB** | **EUCAST MIC breakpoints (mg/L) > R** | **MIC [mg/l]** | | | | | | | |  |
| --- | --- | --- | --- | --- | --- | --- | --- | --- | --- | --- | --- |
|  |  |  | **C20** | **ZnO20** | **ZnONPs 20** | **C40** | **ZnO40** | **ZnONPs40** | **ZnO 20+20** | **ZnONPs 20+20** |  |
|  |  |  |  |  |  |  |  |  |  |  |  |
| **Penicillins** | **AMP** | 8 | 1 | 1 | 1 | 1 | 1 | 1 | 1 | 1 |  |
|  | **SAM** | 8 | 1/0.5 | 1/0.5 | 1/0.5 | 1/0.5 | 1/0.5 | 1/0.5 | 1/0.5 | 1/0.5 |  |
|  | **PIP** | 8 | 1 | 1 | 1 | 1 | 1 | 1 | 1 | 1 |  |
|  | **TZP** | 8 | 1/4 | 1/4 | 1/4 | 1/4 | 1/4 | 1/4 | 1/4 | 1/4 |  |
| **Monobactams** | **ATM** | 4 | 0.125 | 0.125 | 0.125 | 0.125 | 0.125 | 0.125 | 0.125 | 0.125 |  |
| **Cephalosporines** | **CFZ** | 4 | 2 | 4 | 2 | 2 | 4 | 2 | 2 | 2 |  |
|  | **CXM** | 8 | 0.5 | 0.5 | 0.5 | 0.5 | 0.5 | 0.5 | 0.5 | 0.5 |  |
|  | **CTX** | 1 | 0.06 | 0.06 | 0.06 | 0.06 | 0.06 | 0.06 | 0.06 | 0.06 |  |
|  | **CTZ** | 4 | 0.25 | 0.5 | 0.25 | 0.25 | 0.5 | 0.25 | 0.25 | 0.25 |  |
|  | **CFP** | 2 | 0.5 | 0.5 | 0.5 | 0.5 | 0.5 | 0.5 | 0.5 | 0.5 |  |
|  | **SCF** | 2 | 0.5/0.25 | 0.5/0.25 | 0.5/0.25 | 0.5/0.25 | 0.5/0.25 | 0.5/0.25 | 0.5/0.25 | 0.5/0.25 |  |
|  | **FEP** | 4 | 0.12 | 0.12 | 0.12 | 0.12 | 0.12 | 0.12 | 0.12 | 0.12 |  |
| **Aminoglycosides** | **GEN** | 2 | 1 | 2 | 0.5 | 1 | 2 | 0.5 | 1 | 0.5 |  |
|  | **AMK** | 8 | 2 | 8 | 1 | 2 | 8 | 1 | 4 | 1 |  |
|  | **NTL** | 2 | 0.25 | 1 | 0.25 | 0.25 | 1 | 0.25 | 0.25 | 0.25 |  |
|  | **TOB** | 2 | 0.5 | 1 | 0.5 | 0.5 | 1 | 0.5 | 0.5 | 0.5 |  |
| **Sulfonamides** | **SXT** | 4 | 0.03/0.6 | 0.06/1.2 | 0.03/0.6 | 0.03/0.6 | 0.06/1.2 | 0.03/0.6 | 0.03/0.6 | 0.03/0.6 |  |
| **Fluoroquinolones** | **CIP** | 0.5 | 0.06 | 0.06 | 0.06 | 0.06 | 0.06 | 0.06 | 0.06 | 0.06 |  |
| **Carbapenems** | **MEM** | 8 | 0.12 | 0.12 | 0.12 | 0.12 | 0.12 | 0.12 | 0.12 | 0.12 |  |
|  | **ETP** | 0.5 | 0.015 | 0.015 | 0.015 | 0.015 | 0.015 | 0.015 | 0.015 | 0.015 |  |
| **Tetracyclines** | **TGC** | 0.5 | 0.06 | 0.06 | 0.06 | 0.06 | 0.06 | 0.06 | 0.06 | 0.06 |  |
|  | **TCN** | 8 | 0.5 | 0.5 | 0.5 | 0.5 | 0.5 | 0.5 | 0.5 | 0.5 |  |
| **Amphenicols** | **CHL** | 8 | 0.5 | 0.5 | 1 | 0.5 | 0.5 | 1 | 0.5 | 0.5 |  |
| **Peptides** | **CST** | 2 | 0.25 | 0.25 | 0.25 | 0.25 | 0.25 | 0.25 | 0.25 | 0.25 |  |
