# Supplementary material for: Zinc effects on bacteria: insights from Escherichia coli by multi-omics approach
Source: mSystems. 2023 Oct 31;8(6):e00733-23. doi: 10.1128/msystems.00733-23 (PMC10734530; doi:10.1128/msystems.00733-23)
Supplement: Table S2 — Identified plasmids and resistance genes in all tested strains and identified intergenic mutation. [file msystems.00733-23-s0007.docx]

**Table S2** List of identified plasmids and resistance genes in all tested strains (C40, ZnO40, ZnONPs40, ZnO20+20, ZnONPs20+20) (A). List of identified intergenic mutations for ZnO40, ZnONPs40, ZnO20+20 and ZnONPs20+20 treatments (B).

A

| Strain ID | ST^1^ | PlasmidFinder | | Resfinder | | Accession number^2^ |
| --- | --- | --- | --- | --- | --- | --- |
|  |  | IncX4 | Col(pHAD28) | *mdf*(A) | *sitABCD* |  |
| C40 | 73 |  |  |  |  | SRR22878303 |
| ZnO20+20 | 73 |  |  |  |  | SRR22878291 |
| ZnO40 | 73 |  |  |  |  | SRR22878302 |
| ZnONPs20+20 | 73 |  |  |  |  | SRR22878289 |
| ZnONPs40 | 73 |  |  |  |  | SRR22878290 |
| The coloured squares represent the presence of plasmid replicons (blue) and resistance genes (dark red) with coverage and identity above 90%. ^1^ST stands for sequence type, ^2^accession number of genomic reads available in SRA archive | | | | | | |

B

| **Treated strain** | **Mutation type^1^** | **Original codon/base (AA)^2^** | **Alternative codon/base (AA)^3^** | **SNP Location on contig^4^** | **Genes** | **Affected product^5^** | **Product location on contig^6^** | **GO terms BP^7^** |
| --- | --- | --- | --- | --- | --- | --- | --- | --- |
| ZnO40 | SNP | ACC (T) | CCC (P) | 30009 | *mltF* | Membrane-bound lytic murein transglycosylase MltF | 30162-28606 | cell wall macromolecule catabolic process, cell wall organization, peptidoglycan catabolic process |
|  | SNP | TAG (*) | CAG (Q) | 49777 | *rpoS* | RNA polymerase sigma factor RpoS | 49951-49775 | positive regulation of single-species biofilm formation on inanimate substrate, regulation of cell motility, regulation of cellular response to heat and oxidative stress, regulation of response to salt stress |
|  | SNP | CTG (L) | CTT (L) | 88393 | *topA* | Type I DNA topoisomerase | 89784-87187 | metal ion binding |
|  | SNP | GTT (V) | ATT (I) | 50610 | *ins1* | IS1 transposase | 50502-51195 | DNA recombination, transposition |
|  | SNP | CTG (L) | CCG (P) | 10048 | *cpxA* | Envelope stress sensor histidine kinase CpxA | 10565-9192 | cell adhesion involved in biofilm formation, cellular response to cell envelope stress |
|  | SNP | AAC (N) | AAT (N) | 429 |  | Hypothetical protein | 620-1 |  |
| ZnONPs40 | SNP | GTA (V) | GTC (V) | 99400 | *tRNA-Leu* | tRNA-Leu | 99317-99403 | protein biosynthesis |
|  | SNP | TAG (*) | CAG (Q) | 49777 | *rpoS* | RNA polymerase sigma factor RpoS | 49951-49775 | positive regulation of single-species biofilm formation on inanimate substrate, regulation of cell motility, regulation of cellular response to heat and oxidative stress, regulation of response to salt stress |
|  | SNP | TTA (L) | TCA (S) | 19732 | *cdsA* | Phosphatidate cytidylyltransferase CdsA | 20417-19560 | lipid biosynthesis and metabolism, phospholipid biosynthesis and metabolism |
|  | SNP | TTC (F) | GTC (V) | 20336 | *cdsA* | Phosphatidate cytidylyltransferase CdsA | 20417-19560 | lipid biosynthesis and metabolism, phospholipid biosynthesis and metabolism |
|  | Deletion |  | - 5584 bp |  | *vgrG*_1 *- vgrG*_2 | T6SS protein VgrG (two copies), T6SS PAAR protein, hypothetical proteins |  | cytolysis, bacteriocin, hydrolase |
| ZnO20+20 | SNP | GGC (G) | GAC (D) | 115147 | *deaD* | ATP-dependent RNA helicase DeaD | 113613-115502 | processes at low temperatures, cold-shock degradosome with Rnase E |
|  | SNP | GGT (G) | TGT (C) | 63243 | *cysJ* | NADPH-dependent assimilatory sulfite reductase flavoprotein subunit CysJ | 64878-63079 | FMN binding, sulfite reductase (NADPH) activity, cysteine biosynthesis |
|  | SNP | GTG (V) | ATG (M) | 49967 | *qorB* | NAD(P)H:quinone oxidoreductase QorB | 50507-49647 | oxidoreductase activity |
| ZnONPs20+20 | SNP | ATT (I) | GTT (V) | 126557 | *hns* | DNA-binding transcriptional regulator H-NS |  | stress response |
| ^1^Detected mutation type where SNP represents single nucleotide polymorphism |  |  |  |  |  |  |  |  |
| ^2^Original codon (if present in coding sequence) or base (if intergenic) in C40 with aminoacid (AA) change for coding sequences | | | | | | |  |  |
| ^3^Alternative codon (if present in coding sequence) or base (if intergenic) in a treated strain with aminoacid (AA) change for coding sequences | | | | | | |  |  |
| ^4^Location of SNP or product in short-read assembly | | | |  |  |  |  |  |
| ^5^Product(s) affected by the mutation | | |  |  |  |  |  |  |
| ^6^Product(s) location on contig | | |  |  |  |  |  |  |
| ^7^Biological process in GO terms specified in UniProt database, accessed 3.2.2023 | | | | | |  |  |  |
