# Supplementary material for: Zinc effects on bacteria: insights from Escherichia coli by multi-omics approach
Source: mSystems. 2023 Oct 31;8(6):e00733-23. doi: 10.1128/msystems.00733-23 (PMC10734530; doi:10.1128/msystems.00733-23)
Supplement: Table S3 — Heatmaps of Pearson correlation values for each bacterial strain. [file msystems.00733-23-s0008.docx]

**Table S3** Heatmaps of Pearson Correlation Values for each bacterial strain. For transcriptomic data (A), calculated from RPKM values. For proteomic data (B), calculated from protein abundances. Average for each treatment´s Pearson Correlation Values with calculated SEM values are shown for transcriptome (C) and proteome (D).

A B


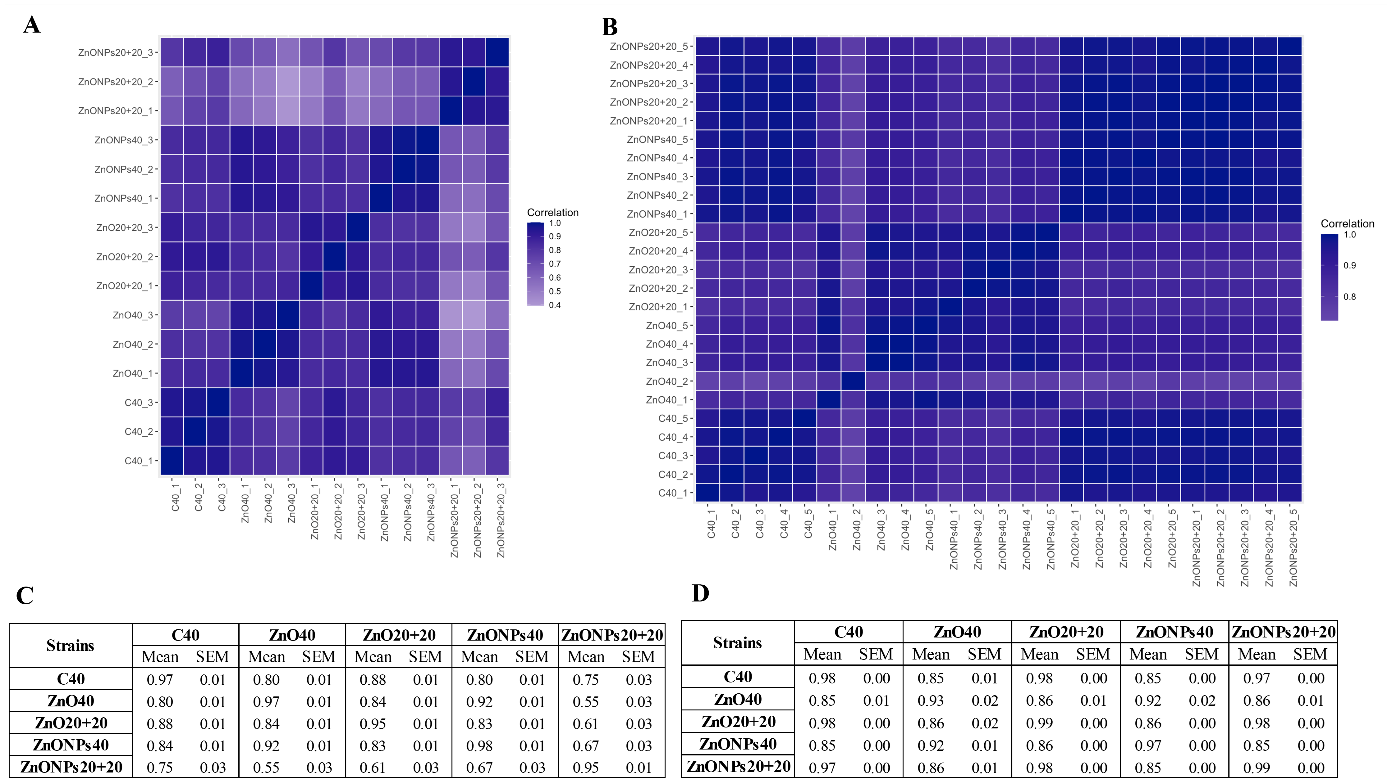

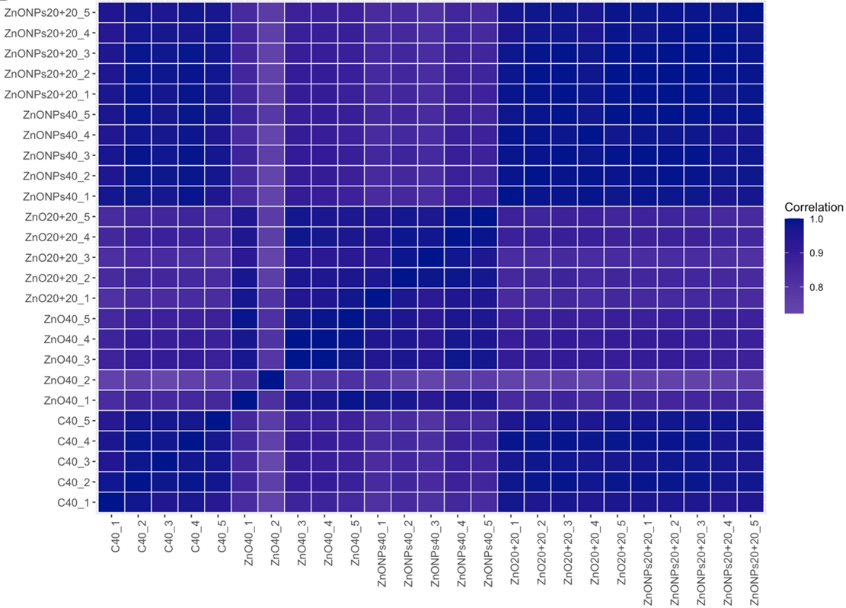


C

| **Strains** | **C40** | | **ZnO40** | | **ZnO20+20** | | **ZnONPs40** | | **ZnONPs20+20** | |
| --- | --- | --- | --- | --- | --- | --- | --- | --- | --- | --- |
|  | Mean | SEM | Mean | SEM | Mean | SEM | Mean | SEM | Mean | SEM |
| **C40** | 0.97 | 0.01 | 0.80 | 0.01 | 0.88 | 0.01 | 0.80 | 0.01 | 0.75 | 0.03 |
| **ZnO40** | 0.80 | 0.01 | 0.97 | 0.01 | 0.84 | 0.01 | 0.92 | 0.01 | 0.55 | 0.03 |
| **ZnO20+20** | 0.88 | 0.01 | 0.84 | 0.01 | 0.95 | 0.01 | 0.83 | 0.01 | 0.61 | 0.03 |
| **ZnONPs40** | 0.84 | 0.01 | 0.92 | 0.01 | 0.83 | 0.01 | 0.98 | 0.01 | 0.67 | 0.03 |
| **ZnONPs20+20** | 0.75 | 0.03 | 0.55 | 0.03 | 0.61 | 0.03 | 0.67 | 0.03 | 0.95 | 0.01 |

D

| **Strains** | **C40** | | **ZnO40** | | **ZnO20+20** | | **ZnONPs40** | | **ZnONPs20+20** | |
| --- | --- | --- | --- | --- | --- | --- | --- | --- | --- | --- |
|  | Mean | SEM | Mean | SEM | Mean | SEM | Mean | SEM | Mean | SEM |
| **C40** | 0.98 | 0.00 | 0.85 | 0.01 | 0.98 | 0.00 | 0.85 | 0.00 | 0.97 | 0.00 |
| **ZnO40** | 0.85 | 0.01 | 0.93 | 0.02 | 0.86 | 0.01 | 0.92 | 0.02 | 0.86 | 0.01 |
| **ZnO20+20** | 0.98 | 0.00 | 0.86 | 0.02 | 0.99 | 0.00 | 0.86 | 0.00 | 0.98 | 0.00 |
| **ZnONPs40** | 0.85 | 0.00 | 0.92 | 0.01 | 0.86 | 0.00 | 0.97 | 0.00 | 0.85 | 0.00 |
| **ZnONPs20+20** | 0.97 | 0.00 | 0.86 | 0.01 | 0.98 | 0.00 | 0.85 | 0.00 | 0.99 | 0.00 |
