# Supplementary material for: Zinc effects on bacteria: insights from Escherichia coli by multi-omics approach
Source: mSystems. 2023 Oct 31;8(6):e00733-23. doi: 10.1128/msystems.00733-23 (PMC10734530; doi:10.1128/msystems.00733-23)
Supplement: Table S4 — Overview of numbers and percentage representation of transcripts and proteins with distribution according to significance and up/downregulation. [file msystems.00733-23-s0009.docx]

Table S4 Overview of numbers and percentage representation of transcripts (A) and proteins (B) with distribution according to significance and up/down-regulation.

| **Transcripts** | **ZnO40** | **ZnONPs 40** | **ZnO 20+20** | **ZnONPs 20+20** |
| --- | --- | --- | --- | --- |
| Total number | 4079 | 4090 | 4062 | 4099 |
| Total number of significant transcripts | 1430 | 1198 | 934 | 1556 |
| Percentage of significant transcripts | 35 | 29 | 23 | 38 |
| Total number of significant DEGs | 296 | 184 | 90 | 238 |
| Significant up-regulated transcripts | 277 | 150 | 73 | 4 |
| Percentage of up-regulated transcripts (from total number) | 7 | 4 | 2 | 0 |
| Percentage of up-regulated transcripts (from total number of significant transcripts) | 19 | 13 | 8 | 0 |
| Significant down-regulated transcripts | 19 | 34 | 17 | 234 |
| Percentage of down-regulated transcripts (from whole number) | 0.5 | 0.8 | 0.4 | 5.7 |
| Percentage of down-regulated transcripts (from total number of significant transcripts) | 1.3 | 2.8 | 1.8 | 15.0 |
|  |  |  |  |  |
| **Proteins** | **ZnO40** | **ZnONPs 40** | **ZnO 20+20** | **ZnONPs 20+20** |
| Total number | 2494 | 2493 | 2497 | 2484 |
| Total number of significant proteins | 232 | 216 | 210 | 231 |
| Percentage of significant proteins | 9 | 9 | 8 | 9 |
| Total number of significant DAPs | 232 | 216 | 156 | 178 |
| Significant up-regulated proteins | 168 | 167 | 109 | 138 |
| Percentage of up-regulated proteins (from total number) | 7 | 7 | 4 | 6 |
| Percentage of up-regulated proteins (from total number of significant proteins) | 72 | 77 | 52 | 60 |
| Significant down-regulated proteins | 64 | 49 | 47 | 40 |
| Percentage of down-regulated proteins (from whole number) | 2.6 | 2.0 | 1.9 | 1.6 |
| Percentage of down-regulated proteins (from total number of significant proteins) | 27.6 | 22.7 | 22.4 | 17.3 |
